# Supplementary material for: Sphingomyelinase Disables Inactivation in Endogenous PIEZO1 Channels
Source: Cell Rep. 2020 Oct 6;33(1):108225. doi: 10.1016/j.celrep.2020.108225 (PMC7539531; doi:10.1016/j.celrep.2020.108225)
Supplement: Document S1. Figures S1–S7 [file mmc1.pdf]

Cell Reports, Volume *33*

Supplemental Information

Sphingomyelinase Disables Inactivation in Endogenous PIEZO1 Channels

Jian Shi, Adam J. Hyman, Dario De Vecchis, Jiehan Chong, Laeticia Lichtenstein, T. Simon Futers, Myriam Rouahi, Anne Negre Salvayre, Nathalie Auge, Antreas C. Kalli, and David J. Beech

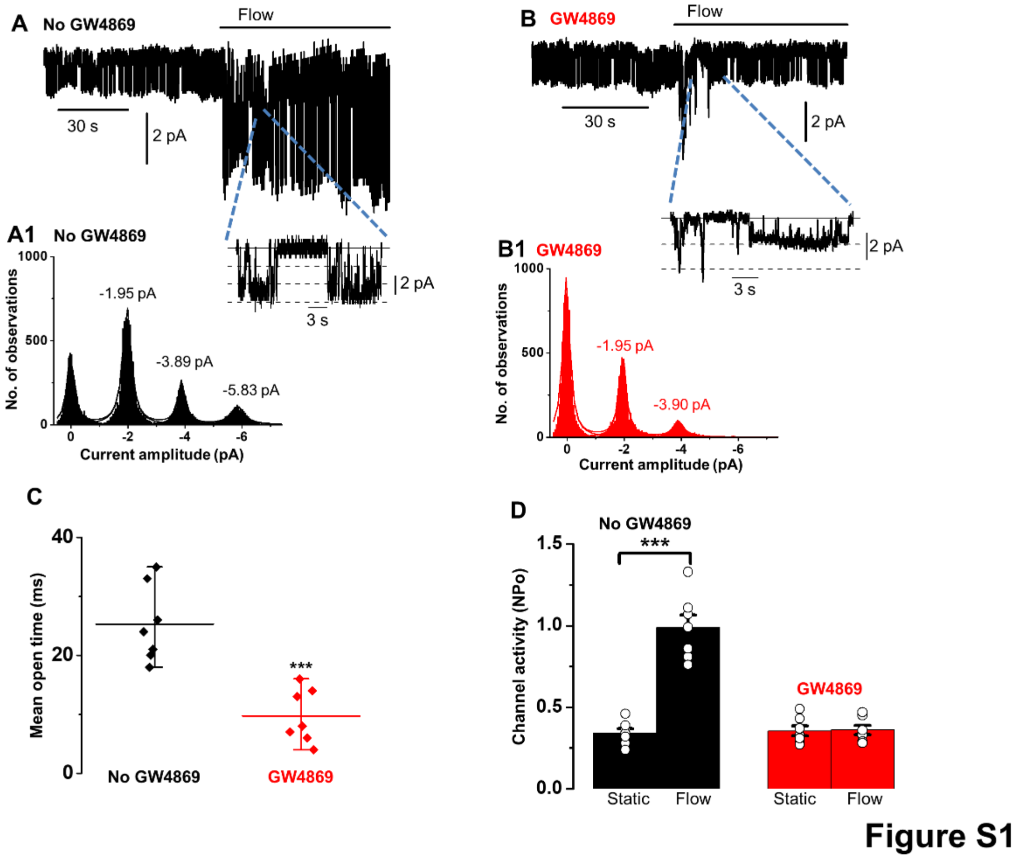

Figure S1. GW4869 Prevents Sustained Channel Activation by Flow. (**A**, **B**) Example traces for PIEZO1 channel currents in outside-out patches voltage-clamped at -80 mV. Constitutive channel activity was observed prior to application of flow at 20 µL.s^{-1}, which stimulated multiple channel openings. (**A**) Control condition: vehicle (0.25% DMSO). (**B**) Test condition: 10 µM GW4869. (**A1**, **B1**) Amplitude histograms for flow-evoked channel activity of (**A**) and (**B**). Current amplitudes for peaks of the fitted distributions are indicated. (**C**) Mean ± s.e.mean channel activity data for experiments of the type shown in (**A**, **B**): Control (n=7 recordings, N=3 mice); GW4869 (n=7, N=3). NP_o is the number of channels multiplied by the open probability of the channels. * P < 0.05 by Student's t-test. Related to Figure 3.

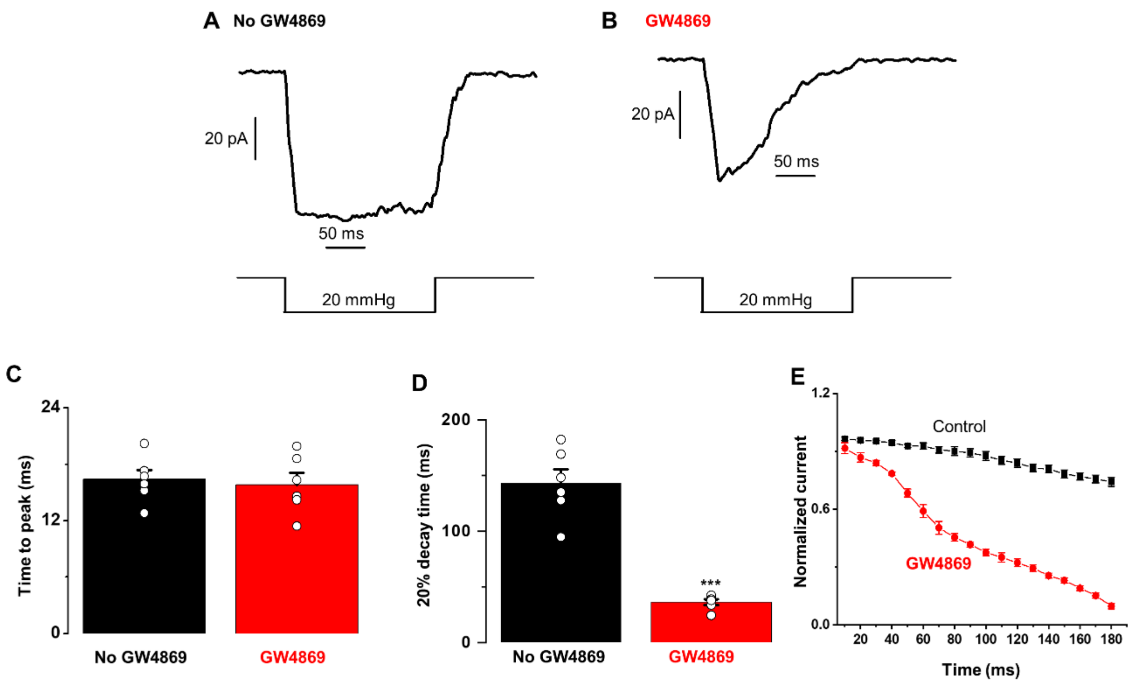

Figure S2

Figure S2. GW4869 Prevents Sustained Channel Activation By Pressure Step. (**A**, **B**) Example traces for macroscopic PIEZO1 channel currents in cell-attached patches voltage-clamped at -80 mV. Negative pressure steps of 20 mmHg were applied to the patch pipette. (**A**) Control condition: vehicle (0.25% DMSO). (**B**) Test condition: 10 µM GW4869. (**C**) Mean ± s.e.mean data for experiments of the type shown in (**A**, **B**), showing the time to reach peak inward current after first applying the pressure step: Control (n=6 recordings, N=3 mice); GW4869 (n=6 recordings, N=3 mice). (**D**) Mean ± s.e.mean data for experiments of the type shown in (**A**, **B**), showing the time for 20% decay of the current after reaching the peak: Control (n=6, N=3); GW4869 (n=6, N=3). *** $P < 0.001$ by Student's t-test. (**E**) As for (**A, B**) showing analysis of the current decay rate for: Control (n=6, N=3) and GW4869 (n=6, N=3) recordings. Related to Figure 4.

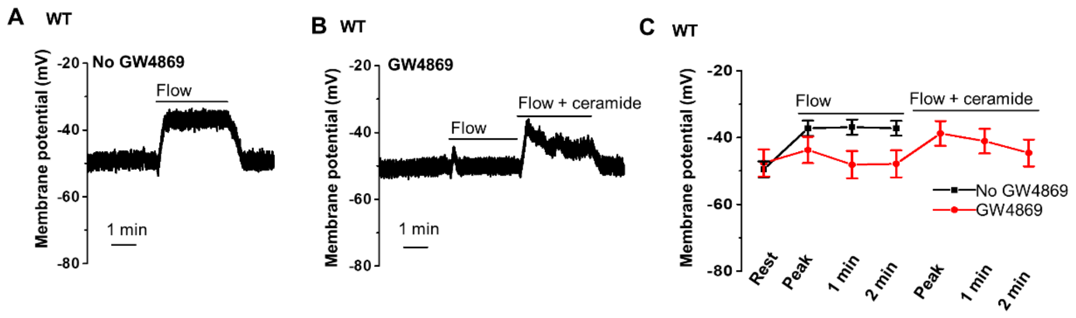

Figure S3

Figure S3. Wildtype Mouse Data For Comparison With Figure 5G-I. All data relate to membrane potential recorded in amphotericin whole-cell mode from freshly isolated endothelium of second-order mesenteric arteries. All data are from endothelium of wildtype (WT) mice. (**A**, **B**) Representative recordings showing responses to 20 µL.s^{-1} fluid flow in the absence and then presence of 10 µM ceramide and in the absence (**A**) and presence (**B**) of 10 µM GW4869. (**C**) Membrane potential mean ± s.e.mean data for experiments of the type shown in (**A**, **B**): n=6 recordings and N=3 mice for both groups. Related to Figure 5.

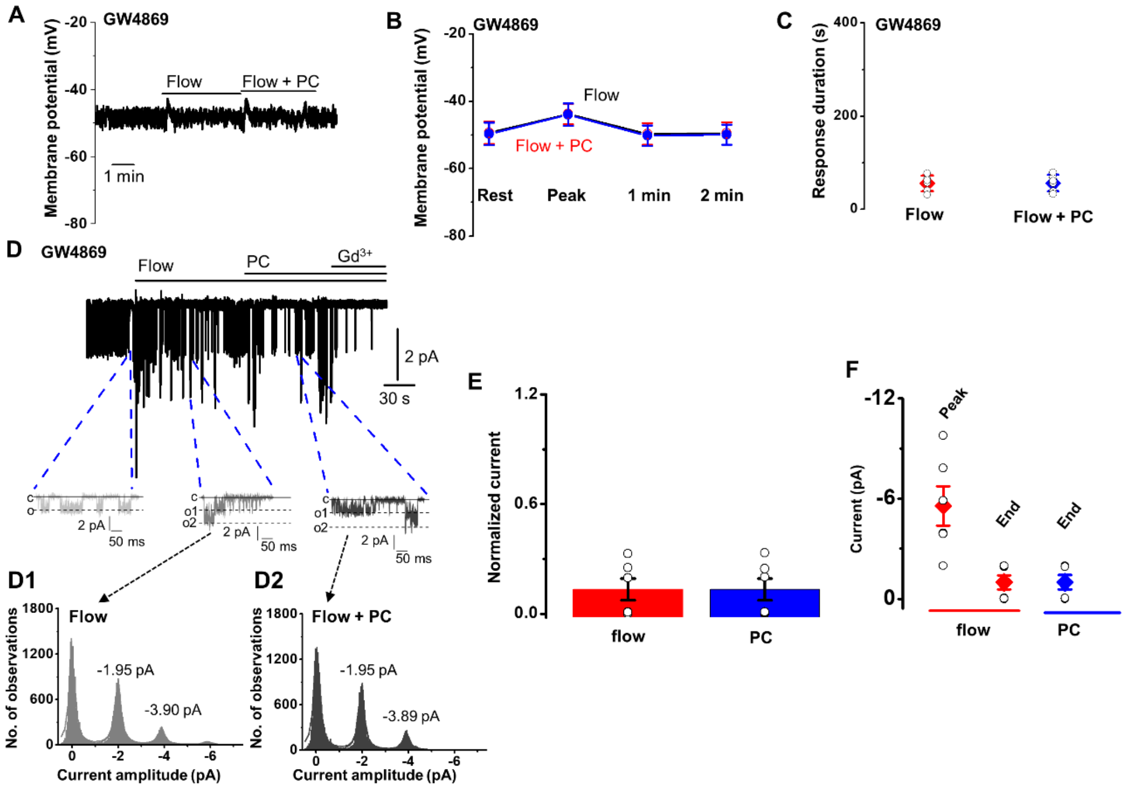

Figure S4

Figure S4. Phosphorylcholine (2-methacryloyloxyethyl PC) Has No Effect. (**A-C**) All data relate to membrane potential recorded in amphotericin whole-cell mode from freshly isolated endothelium of second-order mesenteric arteries. (**A**) Representative recording showing responses to 20 μL.s^{-1} fluid flow in the presence of 10 μM GW4869 in the absence and then presence of 10 μM PC. (**B**) Membrane potential mean ± s.e.mean data for experiments of the type shown in (**A**). (**C**) Response duration mean ± s.e.mean data for experiments of the type shown in (**A**). Original raw data points are superimposed. (**B**, **C**) n=6 recordings, N=3 mice. (**D-F**) All measurements were single channel current recordings from outside-out patches excised from freshly isolated endothelium of second-order mesenteric arteries. Holding potential was -80 mV. (**D**) Representative recording showing response to 20 μL.s^{-1} fluid flow in the presence of 10 μM GW4869 in the absence and then presence of 10 μM PC. Three sections of the traces are shown below on expanded time-scale. (**D1**, **D2**) Amplitude histograms for channel activity in flow and flow plus 2-methacryloyloxyethyl PC, as shown in (**D**). Current amplitudes for peaks of the fitted distributions are indicated. (**E**) For experiments of the type shown in (**D**), all data points and mean ± s.e.mean for the channel currents at the end of recordings with the application of PC (before the addition of Gd^{3+}) normalized to peak currents evoked by flow (n=6 recordings, N=3 mice). (**F**) The values of peak current or the current at the end of recording for each patch shown in (**D**). Related to Figure 6.

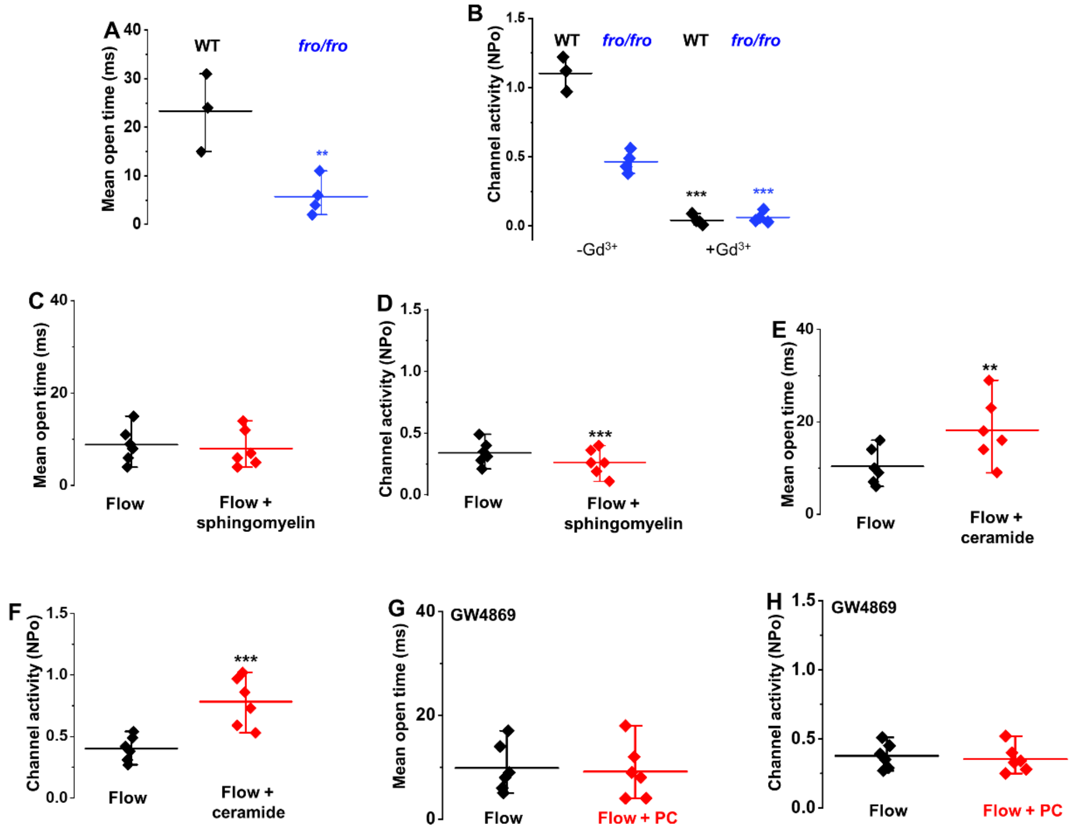

Figure S5

Figure S5. Mean Open Time And Channel Open Probability For The Experiments In Endothelial Cells From WT/ *fro/fro* Mice Or Pre-Treated With Different Lipids. (**A**) Mean open time for experiments of the type shown in **Figure 3A and Figure 3B**, all data points and mean ± s.e.mean for the channel open time 20 s after the initiation of flow (i.e. the sustained response) (WT n=3 recordings, N=3 mice, *fro/fro* n=4, N=3). ** $P < 0.01$. (**B**) Channel open probability (NPo) for experiments of the type shown in **Figure 3** (**A**, **B**, **E**, **F**), all data points and mean ± s.e.mean for channel activity represented by NP_o (channel number multiplied by open probability) measured 20 s after the initiation of flow. Without Gd^{3+} (- Gd^{3+}): WT n=3, N=3; *fro/fro* n=4, N=3. With Gd^{3+} (+ Gd^{3+}): WT n=4, N=3; *fro/fro* n=4, N=3. *** $P < 0.001$. (**C**) Channel open time mean ± s.e.mean data for experiments of the type shown in **Figure 6** (**A**): n=6 recordings, N=3 mice. (**D**) Mean ± s.e.mean channel activity described as NP_o (channel number multiplied by open probability) for experiments of the type shown in **Figure 6** (**A**): n=6 recordings, N=3 mice. (**E**) Channel open time mean ± s.e.mean data for experiments of the type shown in **Figure 6** (**D**): n=6 recordings, N=3 mice. (**F**) Mean ± s.e.mean channel activity described as NP_o for experiments of the type shown in **Figure 6** (**D**): n=6 recordings, N=3 mice. (**G**) Channel open time mean ± s.e.mean data for experiments of the type shown in **SI Figure S4** (**D**): n=6 recordings, N=3 mice. (**H**) Mean ± s.e.mean channel activity described as NP_o (channel number multiplied by open probability) for experiments of the type shown in **SI Figure S4** (**D**): n=6 recordings, N=3 mice. Original raw data points are superimposed. Related to Figure 3.

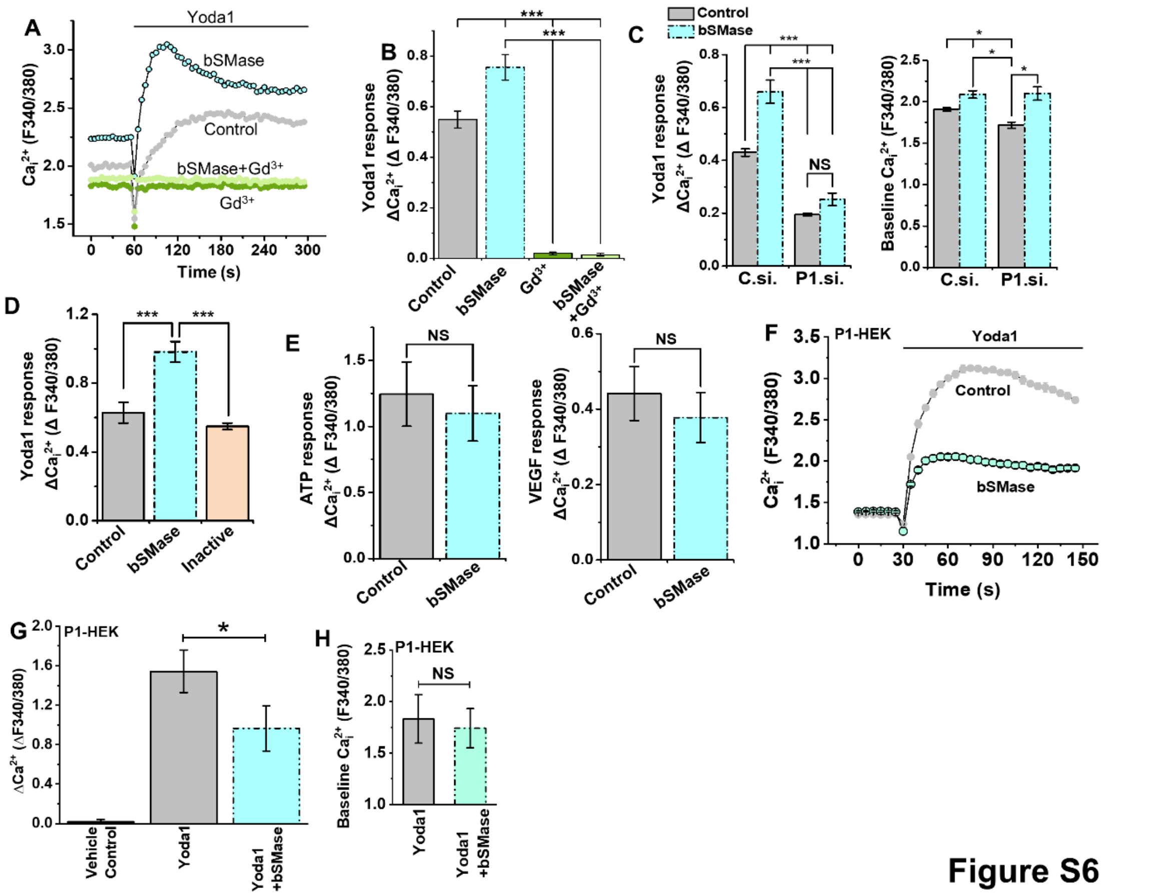

Figure S6

Figure S6. Bacterial Sphingomyelinase Enhances Endothelial PIEZO1 Function But Inhibits PIEZO1 Over-Expressed In HEK 293 Cells. When we first began this project we tested the effects of exogenous bacterial sphingomyelinase (bSMase) on PIEZO1-mediated Ca^{2+} signals evoked by the chemical activator, Yoda1, in cultured human umbilical vein endothelial cells (HUVECs) or HEK 293 cells over-expressing human PIEZO1. Previous studies had described how addition of exogenous sphingomyelinase can modify gating properties of voltage-gated ion channels (Combs et al., 2013). HUVECs were previously shown to express native PIEZO1 (Evans et al., 2018; Li et al., 2014). Data are intracellular Ca^{2+} measurements from HUVECs or from HEK T-REx™ cells overexpressing human PIEZO1 on induction with tetracycline (P1-HEK cells). (**A**) Example time-series traces showing effects of 1 μM Yoda1 on intracellular Ca^{2+} indicated by F340/380 in cells in a single 96-well plate without pre-treatment (Control) or after pre-treatment for 30 min at 37 ˚C with 0.5 $U.mL^{-1}$ bSMase or 100 μM gadolinium chloride (Gd^{3+}) or both. (**B**) As for (**A**) but plotted as the peak change in intracellular Ca^{2+} (ΔCa^{2+}_i) above baseline in response to Yoda1 and showing mean ± s.e.mean data for multiple independent experiments (n/*N*=3/4). (**C**) Mean ± s.e.mean data for ΔCa^{2+}_i in response to 1 μM Yoda1 (left) and baseline Ca^{2+} indicated by basal F340/380 in the same experiments (n/*N*=3/4). C.si. was control siRNA and P1.si. PIEZO1 siRNA. (**D**) Similar to (**B**) but comparing the effect of 30 min pre-treatment with 0.5 $U.mL^{-1}$ bSMase or the same concentration of heat-inactivated bSMase on the effect of 1 μM Yoda1 (n/*N*=3/4). (**E**) Similar to (**B**) but in which Ca^{2+} responses were evoked by 20 μM ATP or 30 $ng.mL^{-1}$ vascular endothelial growth factor (VEGF) in place of Yoda1 (n/*N*=3/4 each). (**F**) Example time-series traces showing effects of 1 μM Yoda1 on intracellular Ca^{2+} indicated by F340/380 in cells without pre-treatment (Control) or after pre-treatment for 30 min at 37 ˚C with 0.5 $U.mL^{-1}$ bSMase. In the absence of exogenous PIEZO1 these cells show no Ca^{2+} response to Yoda1(Evans et al., 2018). (**G**) As for (**F**) but plotted as the peak change in intracellular Ca^{2+} (ΔCa^{2+}_i) above baseline in response to the vehicle control for Yoda1 (DMSO), 1 μM Yoda1 or 1 μM Yoda1 after pre-treatment with bSMase, showing mean ± s.e.mean data for multiple independent experiments (n/*N*=3/4). (**H**) Mean ± s.e.mean data for baseline Ca^{2+} indicated by basal F340/380 in the same experiments as (**G**). *** $P < 0.01$, * $P < 0.05$, NS:not significantly different. Related to Figures 3 and 4.

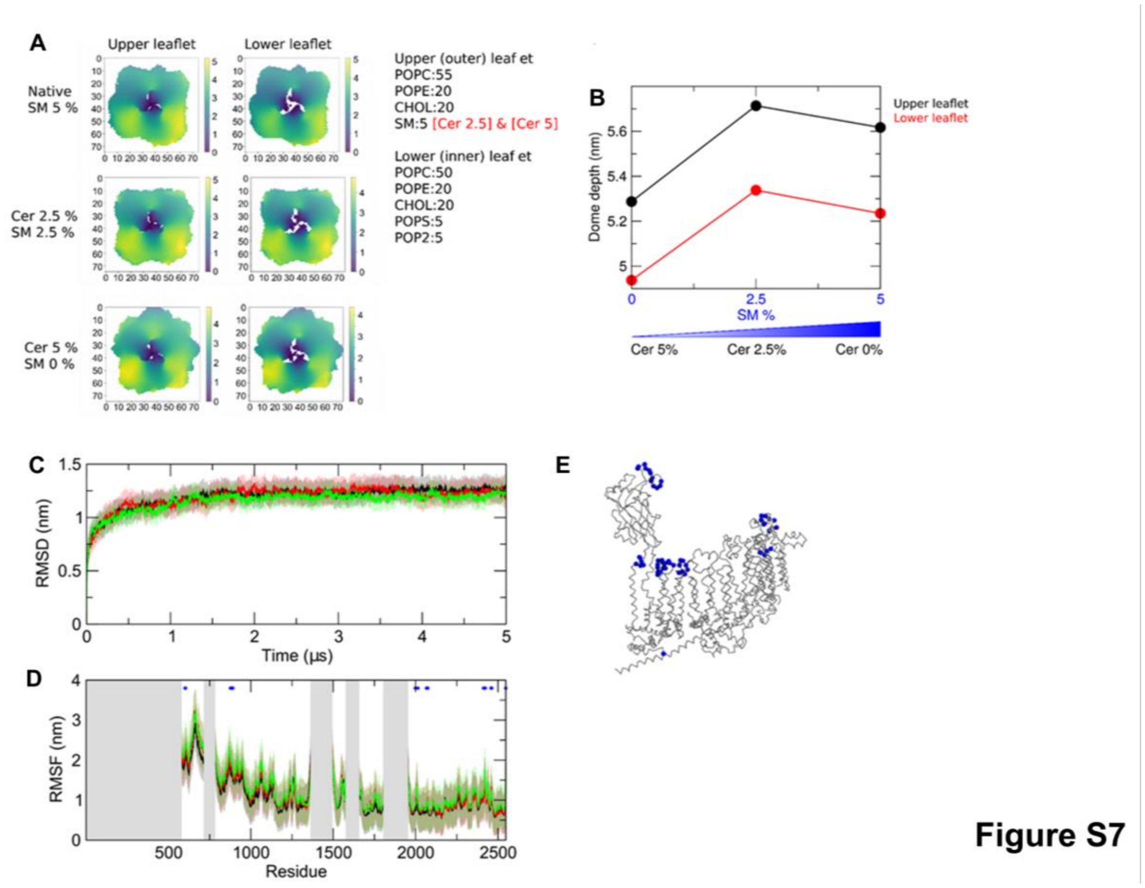

Figure S7. Potential Molecular Mechanism Underlying Regulation Of Piezo1 By Ceramide And Sphingomyelin In Endothelial Cells. To investigate whether the dome could be affected by changes in sphingomyelin and ceramide concentration, we performed molecular dynamics simulations in which the 3D structure of PIEZO1 (Guo and MacKinnon, 2017) was inserted in model asymmetric membranes that resemble the endothelial plasma membrane (Takamura et al., 1990). We included different concentrations of sphingomyelin and ceramide: 5% sphingomyelin, 5% ceramide and a combination at 2.5% each. In all simulations, PIEZO1 created a dome in the membrane with a trilobed topology in good agreement with previous studies (Chong et al., 2019). However, the depth of the dome was lower in the systems in which 5% ceramide was added. Impact of sphingomyelin and ceramide on the PIEZO1 channel dome depth is shown in (A) and (B). Stability and fluctuation analyses of the PIEZO1 structure during the molecular dynamics simulations are shown in (C), (D) and (E). (**A**) Height map of the z-coordinate of the CG phosphate particles corresponding to the upper (left) and lower (right) leaflet, averaged across all repeat simulations of the same simulation system. This analysis is shown for the 3 different simulation systems with different concentrations of sphingomyelin and ceramide. (**B**) Average dome depth of the upper and lower leaflets in the 3 simulation systems. With 2.5% sphingomyelin and 2.5% ceramide, the dome reached maximum depth of 5.71 ± 0.00778 nm and 5.34 ± 0.00847 nm for the upper and lower leaflets. With 5% sphingomyelin the depth was 5.62 ± 0.00899 nm and 5.23 ± 0.00963 nm for the upper and lower leaflets, whereas with 5% ceramide it was 5.29 ± 0.00708 nm and 4.94 ± 0.00767 nm. All phospholipids and the sphingomyelin remained in the leaflet of their origin at the beginning of the simulations. Ceramide and cholesterol were seen to flip-flop between the leaflets. (**C**) Root mean square deviation (RMSD) of the protein as a function of time in the simulated systems. The RMSD of the protein for the simulations with the native bilayer is shown in black, for the bilayer with 2.5% ceramide is shown in red, and for the membrane with 5% ceramide is shown in green. (**D**) Root mean-square fluctuation (RMSF) of the protein calculated in the simulated systems. The color code is the same as in (**C**). Blue spheres show the residues that are not present in the PIEZO1 cryo-EM structure that have been added during the modelling (see STAR Methods). (**E**) Last frame of one of the coarse-grained simulations showing a single chain from the PIEZO1 structure (gray). The residues in blue are the residues that are missing from the cryo-EM structure that were added during the modelling stage (see STAR Methods). Related to Figure 7.
