# Supplementary material for: Sphingomyelinase Disables Inactivation in Endogenous PIEZO1 Channels
Source: Cell Rep. 2020 Oct 6;33(1):108225. doi: 10.1016/j.celrep.2020.108225 (PMC7539531; doi:10.1016/j.celrep.2020.108225)
Supplement: Document S2. Article plus Supplemental Information [file mmc2.pdf]

Cell Reports

Article

Sphingomyelinase Disables Inactivation in Endogenous PIEZO1 Channels

Graphical Abstract

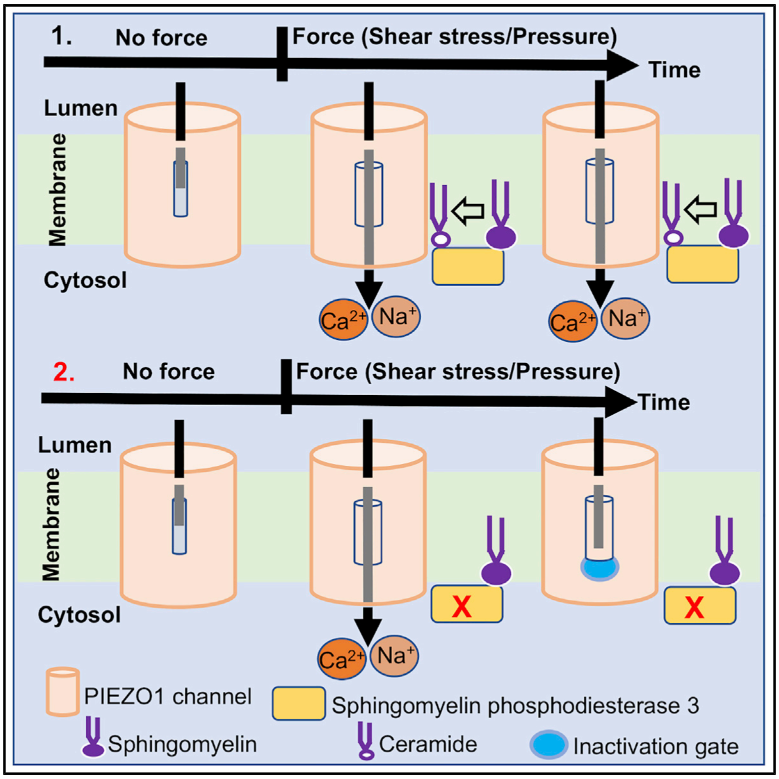

Highlights

- SMPD3 sphingomyelinase enables long-lasting PIEZO1 activity in response to force
- Ceramide, a key lipid product of SMPD3, promotes long-lasting activity
- Sphingomyelin, the SMPD3 substrate, does not affect the duration of activity
- Sphingomyelin alters PIEZO1 force sensitivity

Authors

Jian Shi, Adam J. Hyman, Dario De Vecchis, ..., Nathalie Auge, Antreas C. Kalli, David J. Beech

Correspondence

j.shi1@leeds.ac.uk (J.S.), a.kalli@leeds.ac.uk (A.C.K.), d.j.beech@leeds.ac.uk (D.J.B.)

In Brief

The vascular endothelial response to blood flow involves sustained activation of mechanically activated PIEZO1 channels; yet, PIEZO1 has an intrinsic rapid inactivation gate that should prevent long-lasting activity. Shi et al. show that the gate is disabled in the endothelium by sphingomyelinase that produces ceramide, thus enabling physiologically appropriate responses.

Shi et al., 2020, Cell Reports *33*, 108225
October 6, 2020 © 2020 The Authors.
https://doi.org/10.1016/j.celrep.2020.108225

CellPress

Cell Reports

CellPress
OPEN ACCESS

Article

Sphingomyelinase Disables Inactivation in Endogenous PIEZO1 Channels

Jian Shi,[1,4,*] Adam J. Hyman,[1] Dario De Vecchis,[1] Jiehan Chong,[1] Laeticia Lichtenstein,[1] T. Simon Futers,[1] Myriam Rouahi,[3] Anne Negre Salvayre,[3] Nathalie Auge,[3] Antreas C. Kalli,[1,2,*] and David J. Beech[1,*]
[1]Leeds Institute of Cardiovascular and Metabolic Medicine, School of Medicine, University of Leeds, Leeds LS2 9JT, UK
[2]Astbury Centre for Structural Molecular Biology, University of Leeds, Leeds LS2 9JT, UK
[3]INSERM U-1048 and Université Paul Sabatier, 31432 Cedex 4 Toulouse, France
[4]Lead Contact
*Correspondence: j.shi1@leeds.ac.uk (J.S.), a.kalli@leeds.ac.uk (A.C.K.), d.j.beech@leeds.ac.uk (D.J.B.)
https://doi.org/10.1016/j.celrep.2020.108225

SUMMARY

Endogenous PIEZO1 channels of native endothelium lack the hallmark inactivation often seen when these channels are overexpressed in cell lines. Because prior work showed that the force of shear stress activates sphingomyelinase in endothelium, we considered if sphingomyelinase is relevant to endogenous PIEZO1. Patch clamping was used to quantify PIEZO1-mediated signals in freshly isolated murine endothelium exposed to the mechanical forces caused by shear stress and membrane stretch. Neutral sphingomyelinase inhibitors and genetic disruption of sphingomyelin phosphodiesterase 3 (SMPD3) cause PIEZO1 to switch to profoundly inactivating behavior. Ceramide (a key product of SMPD3) rescues non-inactivating channel behavior. Its co-product, phosphoryl choline, has no effect. In contrast to ceramide, sphingomyelin (the SMPD3 substrate) does not affect inactivation but alters channel force sensitivity. The data suggest that sphingomyelinase activity, ceramide, and sphingomyelin are determinants of native PIEZO gating that enable sustained activity.

INTRODUCTION

Shear stress is a frictional force that arises when fluid flows along a cellular surface: it impacts many aspects of biology (Mammoto and Ingber, 2010). Especially rapid flow occurs in the vasculature where powerful shear stress arises from blood or lymph flowing against the endothelium (Chiu and Chien, 2011). The ability of endothelial cells to sense this force and respond appropriately is critical in vascular development, health, and disease (Chiu and Chien, 2011; Mammoto and Ingber, 2010). Yet, the underlying molecular mechanisms (particularly how the force is sensed) have been difficult to elucidate (Baratchi et al., 2017; Chiu and Chien, 2011). Recently, an important molecular component was revealed as the PIEZO1 channel (Beech and Kalli, 2019; Li et al., 2014), which is a Ca^{2+}-permeable non-selective cationic channel that seems to have as its primary function the sensing of mechanical force and its transduction into cellular response (Beech and Kalli, 2019; Coste et al., 2010; Douguet et al., 2019; Honoré et al., 2015; Murthy et al., 2017). Endothelial PIEZO1 has been found to be important in numerous vascular and other cardiovascular biology that depends on mechanical force (Beech and Kalli, 2019), including vascular maturation in the embryo (Li et al., 2014), blood pressure regulation (Rode et al., 2017), vascular permeability (Friedrich et al., 2019), atherosclerosis (Albarrán-Juárez et al., 2018), response to shear stress in endothelial cells *in vitro* and *in vivo* (Beech and Kalli, 2019; Li et al., 2014; Rode et al., 2017), lymphatic structure, and the disease of generalized lymphatic dysplasia (Fotiou et al., 2015). It alone confers shear stress response on otherwise resistant cells and responds quickly and reversibly in membrane patches excised from endothelial cells, suggesting a membrane-delimited, perhaps direct, ability to sense shear stress (Beech and Kalli, 2019; Li et al., 2014; Rode et al., 2017). It does not inactivate in response to sustained flow and so impacts continuously until flow ceases (Li et al., 2014; Rode et al., 2017). Here, we focus on the non-inactivating property because it contrasts with the rapid complete inactivation often seen when PIEZO1 is overexpressed in cell lines or other types of cells such as neurons (Coste et al., 2010; Del Mármol et al., 2018; Zheng et al., 2019). It raises the possibility that there is a process in endothelial cells that disables the intrinsic fast inactivation gate of PIEZO1 to enable compatibility with the sustained requirements of vascular flow sensing.

A potential explanation for the non-inactivating properties is a factor existing in sufficient quantity relative to PIEZO1 to disable the inactivation gate. We were, therefore, interested in prior vascular studies showing that fluid flow stimulates neutral sphingomyelinase to generate ceramide lipids from the constituent membrane lipid sphingomyelin (Airola and Hannun, 2013; Czarny et al., 2003; Czarny and Schnitzer, 2004).

Neutral and acidic sphingomyelinases of the sphingomyelinase family were previously shown to contribute to the physiology of cardiovascular cell types including endothelial cells (Pavoine and Pecker, 2009). However, only neutral

This is an open access article under the CC BY license (http://creativecommons.org/licenses/by/4.0/).

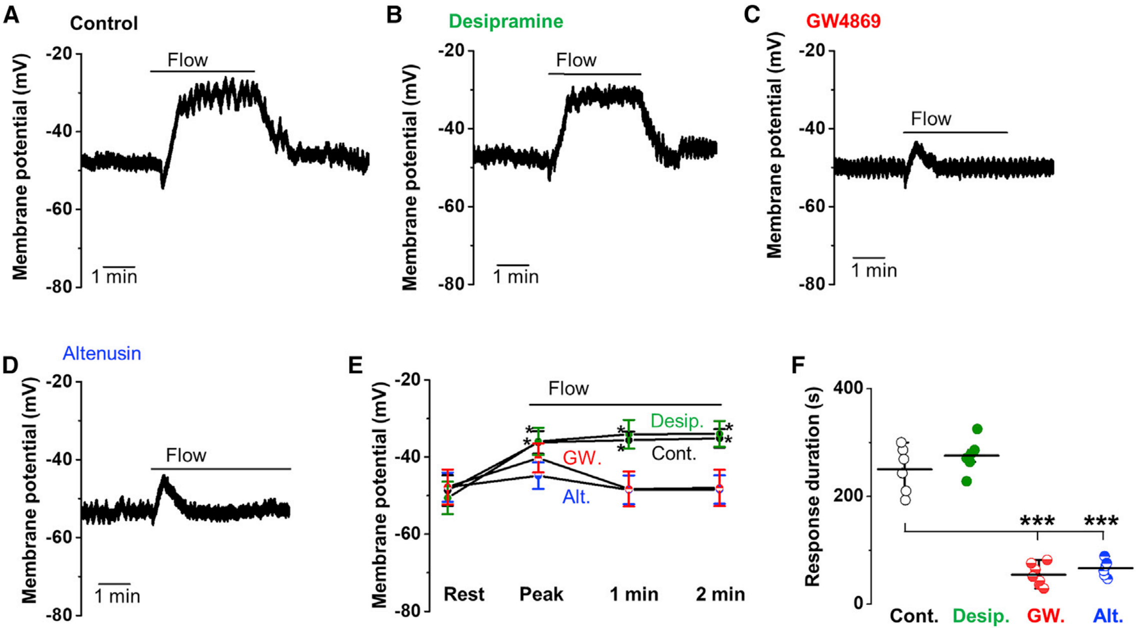

Figure 1. Inhibitors of Neutral Sphingomyelinase Suppress Sustained Flow-Evoked Depolarization

All data relate to the membrane potential recorded in amphotericin whole-cell mode from freshly isolated endothelium of second-order mesenteric arteries.

(A) Representative recording showing the sustained response to application of 20 μL s^{-1} fluid flow in the presence of the vehicle control (0.25% DMSO).

(B–D) Similar to (A) but showing responses after 10-min pre-incubation and the continuous presence of 10 μM desipramine (B), 10 μM GW4869 (C), or 10 μM altenusin (D).

(E) Mean ± SEM data for experiments of the type shown in (A)–(D): control (Cont., n = 6 recordings, N = 3 mice), desipramine (Desip., n = 6, N = 3),; GW4869 (GW., n = 7, N = 3), and altenusin (Alt., n = 6, N = 3). “Rest” indicates the resting membrane potential in static (no flow) condition. “Peak” indicates the maximum depolarization of the membrane potential in response to flow (i.e., the initial response). Membrane potential values 1 and 2 min after the start of flow are also shown.

(F) For the experiments of (E), the duration of the flow-evoked depolarization, which was the time from the onset of flow to when depolarization had faded back to baseline. In Cont. and Desp., the duration of the response was the same as the duration of the exposure to flow because the depolarization did not decay. Mean ± SEM data; *$p < 0.05$, ***$p < 0.001$.

sphingomyelinase has been suggested to be mechanosensitive (Czarny et al., 2003; Czarny and Schnitzer, 2004). Furthermore, the prominent neutral sphingomyelinase sphingomyelin phosphodiesterase 3 (SMPD3 or nSMase2) appears to exert similar functions in endothelial cells as PIEZO1 channels. Both proteins can promote inflammation and atherosclerosis (Albarrán-Juárez et al., 2018; Lallemand et al., 2018), indicating a possible coupling between these two proteins in endothelial cells.

For this reason, we investigated the relevance of sphingomyelinase to endothelial PIEZO1 and sought to determine whether it might explain some of the distinct properties of native endothelial PIEZO1 channels. We focused primarily on using patch-clamp techniques because of their capability to provide high-resolution time-resolved recordings of native PIEZO1 channel activity in response to mechanical stimuli. Our previous work has suggested the effectiveness of this approach when applied to the endothelium of murine mesenteric arteries, in which PIEZO1 channels are the dominant mechanically activated channels (Rode et al., 2017). Thus, we adopted a similar technical approach here. Functional relevance of these channels to exercise-dependent elevation of blood pressure has been suggested by endothelial-specific PIEZO1 disruption in mice (Rode et al., 2017). In this study, we investigated whether neutral sphingomyelinase, particularly SMPD3, regulates PIEZO1 channels and investigated the underlying cellular mechanism in endothelial cells.

RESULTS

Neutral Sphingomyelinase Inhibitors Prevent Sustained Response to Flow

Endothelium was freshly isolated from second-order mesenteric arteries of adult mice and studied acutely without cell or organ culture. Previous membrane potential recordings from this preparation showed an essential role for endogenous PIEZO1 in determining resting membrane potential and sustained depolarization evoked by fluid flow (Rode et al., 2017). Here, we made similar observations, again observing robust reversible depolarization in response to fluid flowing out of a capillary tube (Figure 1A). To test the contribution of endogenous sphingomyelinase, we first incubated cells with pharmacological inhibitors of two different subtypes of this enzyme for 10 mins prior to patch recordings and kept the presence of the inhibitor during recordings. The inhibitor, or its combination with lipid in subsequent studies, was applied using this same protocol, unless stated otherwise. Desipramine, a weak base that inhibits acid

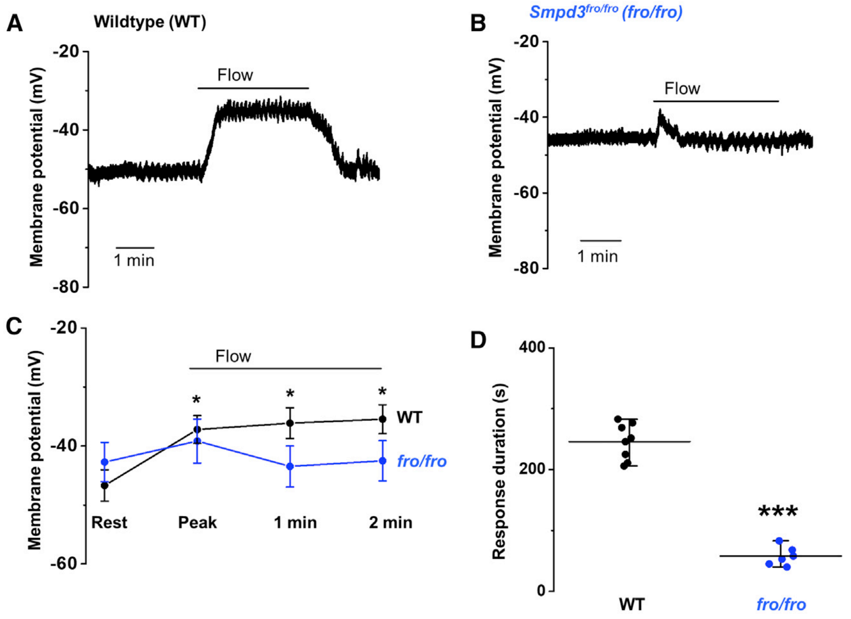

Figure 2. Absence of Sustained Flow-Evoked Depolarization in Smpd3$^{fro/fro}$ Mice

All data relate to membrane potential recorded in amphotericin whole-cell mode from freshly isolated endothelium of second-order mesenteric arteries.

(A) Representative recording showing response to application of 20 μL s^{-1} fluid flow in the endothelium of wild-type (WT) mice.

(B) Representative recording showing response to application of 20 μL s^{-1} fluid flow in the endothelium of *Smpd3$^{fro/fro}$* mice (*fro/fro*).

(C) Mean ± SEM data for experiments of the type shown in (A) and (B). Membrane potential values 1 and 2 min after the start of flow are also shown.

(D) For the experiments of (C), the duration of the flow-evoked depolarization. In the WT group, the duration of the response was the same as the duration of the exposure to flow. Mean ± SEM data; *$p < 0.05$, ***$p < 0.001$. WT: n = 8 recordings, N = 4 mice; *Fro/fro*: n = 6, N = 3.

sphingomyelinase (Kornhuber et al., 2008), had no effect on the flow response, but two inhibitors of neutral sphingomyelinase, GW4869 and altenusin (Airola and Hannun, 2013; Kumar et al., 2019; Luberto et al., 2002), caused flow responses to become transient, such that sustained responses were no longer evident (Figures 1B–1F). There was sustained response only in the vehicle-control condition or the presence of desipramine (Figures 1E and 1F). None of the agents affected the resting membrane potential (Figure 1E). The data suggest that a neutral sphingomyelinase is required for the sustained response to flow.

Disruption of *Smpd3* Prevents Sustained Response to Flow

Small-molecule inhibitors are not necessarily specific and do not shed light on the subtype of neutral sphingomyelinase, so we sought to identify the underlying gene. A prominent neutral sphingomyelinase in vascular biology is SMPD3 or nSMase2 (Pavoine and Pecker, 2009). It is encoded by the *Smpd3* gene, the best characterized gene of the four nSMase genes (Airola and Hannun, 2013; Pavoine and Pecker, 2009). We therefore investigated mice homozygous for the fragilitas ossium (Fro) mutation of the *Smpd3* gene (*Smpd3$^{fro/fro}$*), which causes a loss of the C-terminal active domain of SMPD3 (Airola and Hannun, 2013; Aubin et al., 2005; Lallemand et al., 2018). Although these mice exhibit skeletal dysplasia, they mature to be relatively normal and are suitable for detailed adult cardiovascular phenotyping (Lallemand et al., 2018). Consistent with this relative normality, the resting membrane potential of endothelium from second-order mesenteric artery was similar to that of wild-type (WT) mice (Figures 2A–2C). Strikingly, however, there was an initial transient membrane potential response but no sustained response to flow (Figures 2A–2D). The data suggest that SMPD3 is the neutral sphingomyelinase required for sustained flow-evoked depolarization.

Regulation by SMPD3 Is Local and Membrane Delimited

Sustained activation of PIEZO1 channels is preserved in membrane patches excised from the endothelium, suggesting that intracellular communication and organelles are not required (Rode et al., 2017). All channel activity evoked by fluid flow in these studies was PIEZO1 dependent and had the expected unitary current properties of PIEZO1 channels (Rode et al., 2017). It is also known that SMPD3 is a plasma-membrane-tethered enzyme (Airola and Hannun, 2013). Therefore, we investigated if the SMPD3-PIEZO1 relationship is functional in the excised cell-free membrane by using voltage-clamped outside-out patches and the stimulus of fluid flow out of a capillary tube. *Smpd3$^{fro/fro}$* caused PIEZO1 channels to become strongly inactivating (Figures 3A–3D). Based on unitary current characteristics, the channels were otherwise indistinguishable from those in patches from WT endothelium (Figure 3B1 cf. Figure 3A1). The unitary current size (~1.95 pA at −80 mV) and therefore unitary conductance (~25 pS) were as expected for PIEZO1 channels (Rode et al., 2017). Also consistent with the channels being mediated by PIEZO1, the activity was suppressed but not abolished by Gd^{3+}, which at 10 μM inhibits PIEZO1 channel currents (Coste et al., 2010; Rode et al., 2017; Figures 3E–3H cf. Figures 3A–3D). Gd^{3+} is not a specific PIEZO1 inhibitor. GW4869 similarly suppressed the sustained response of these channels to flow in excised patches (Figure S1). The data suggest that SMPD3 operates locally to suppress inactivation of PIEZO1 channels.

SMPD3 Prevents Pressure-Evoked Inactivation

Activation by flow is physiological but slower in onset than the rapid force caused by pressure pulses and cell prodding used in studies of overexpressed PIEZO1 channels (Coste et al., 2010; Zheng et al., 2019). We therefore also applied pressure pulses to cell-attached patches of endothelium. Similar to previous results in human umbilical vein endothelial cells (HUVECs)

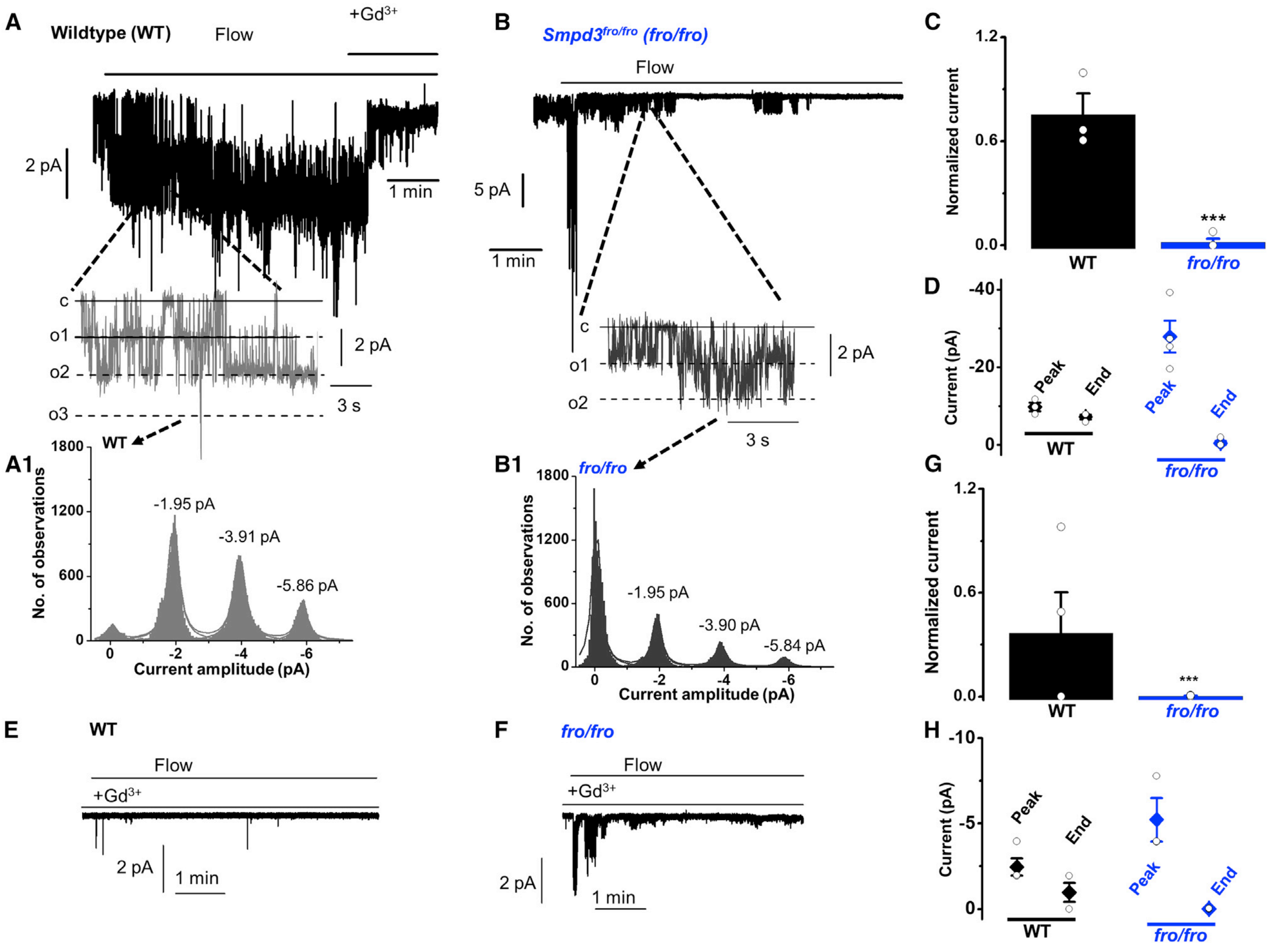

Figure 3. PIEZO1 and SMPD3 Function Together in a Membrane-Delimited Mechanism

All measurements were single-channel current recordings from outside-out patches excised from freshly isolated endothelium of second-order mesenteric arteries. Holding potential was −80 mV.

(A and B) Representative recordings showing responses to 20 μL s^{-1} fluid flow in patches from endothelium of WT mice (A) or *Smpd3*$^{fro/fro}$ (*fro/fro*) mice (B). Note the large initial peak current in the top panel of (B). (A1 and B1) Amplitude histograms for flow-evoked channel activity of (A) and (B). Current amplitudes for peaks of the fitted distributions are indicated.

(C) For experiments of the type shown in (A) and (B), all data points and mean ± SEM for the channel currents at the end of recordings (but before application of Gd^{3+}) normalized to peak currents (WT: n = 3, recordings, N = 3 mice; *fro/fro*: n = 4, N = 3). ***p < 0.001.

(D) The values of peak and end currents for each patch shown in (A) and (B).

(E and F) Representative recordings showing responses to 20 mL.s^{-1} fluid flow in patches from endothelium pretreated with Gd^{3+} of WT mice (E) or *fro/fro* mice (F).

(G) For experiments in the presence of 10 μM Gd^{3+}, of the type exemplified in (E) and (F), all data points and mean ± SEM for the channel currents at the end of recordings normalized to peak currents (WT: n = 4 recordings, N = 3 mice; *fro/fro*: n = 4, N = 3). ***p < 0.001.

(H) The values of peak and end currents for each patch shown in (E) and (F).

(Li et al., 2014), there was sustained channel activation in response to −5 to −40 mmHg pressure pulses applied to patches on freshly isolated endothelium (Figure 4A). The currents increased progressively in response to increased pressure steps and reached a maximum at about −40 mmHg (Figure 4A). They were large multi-channel currents, larger than those seen in outside-out patches (Figure 4A cf. Figure 3A), perhaps at least partly because larger patch pipettes were used for these recordings. The currents were absent in patches from mice that had conditional endothelial-specific deletion of PIEZO1 (PIEZO1$^{\Delta EC}$) (Rode et al., 2017), showing that they depended on PIEZO1 (Figures 4A and 4B). We therefore compared the current kinetics for patches exposed to −20 mmHg pressure from WT and *Smpd3*$^{fro/fro}$ mice (Figures 4C–4F). Strikingly, in patches from *Smpd3*$^{fro/fro}$ mice, the current inactivated, as shown by a strong decay of the inward current despite the persistent pressure step (Figure 4D). Unexpectedly, the initial peak current was larger than the control (Figure 4E), but it then decayed to become much smaller than the control (Figures 4F and 4G). The activation time to reach peak current was not affected in cells from *Smpd3*$^{fro/fro}$ mice (Figure 4H). GW4869 similarly caused the current to inactivate strongly but did not cause an increase in

CellPress
OPEN ACCESS

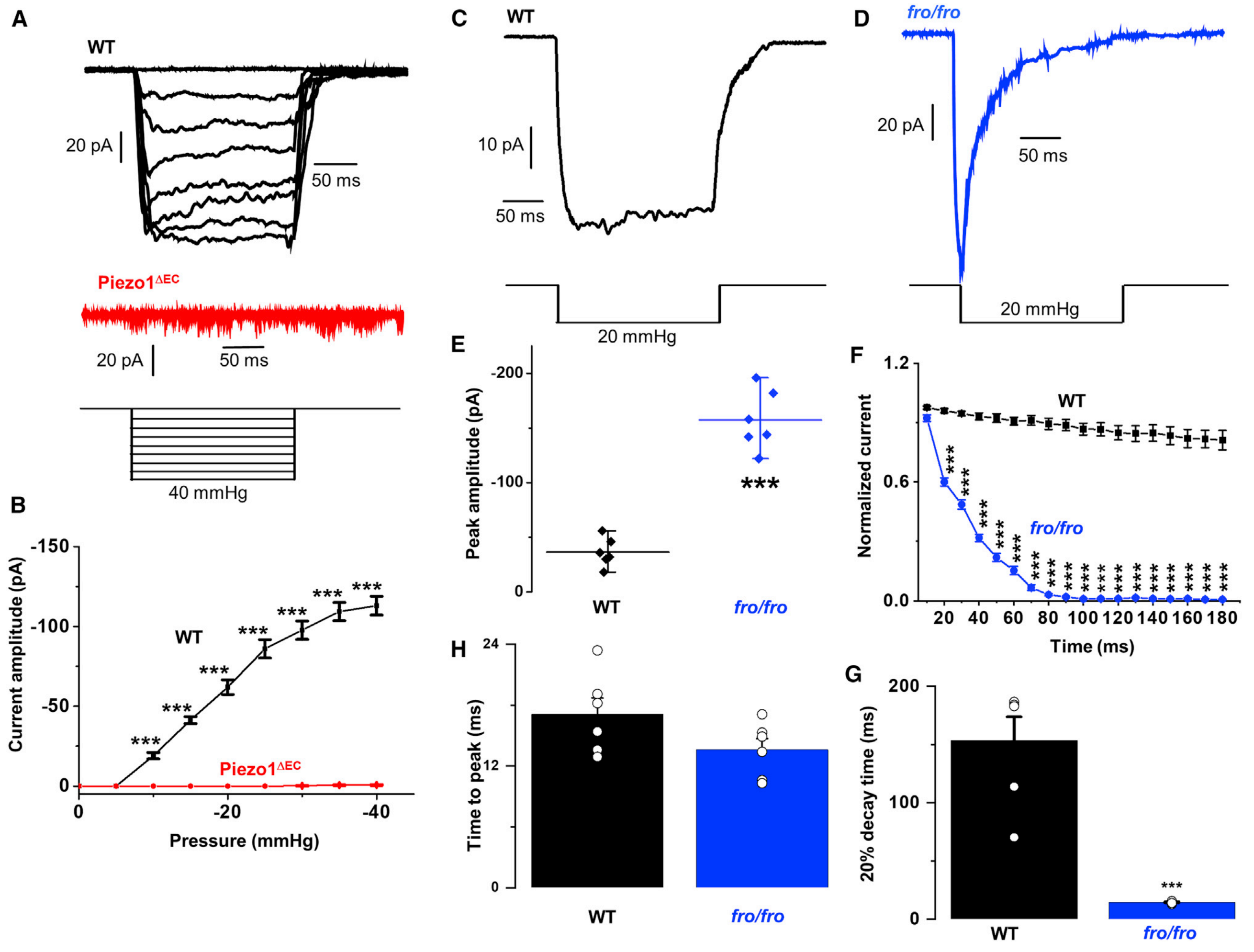

Figure 4. SMPD3 Is Also Important when the Stimulus Is a Pressure Pulse

All measurements were made from cell-attached patches on freshly isolated endothelium of second-order mesenteric arteries of adult mice.

(A) Representative currents elicited by increasing pressure steps from −5 to −40 mmHg, as illustrated schematically in the bottom panel. The top set of currents was from a WT mouse and the set below from a PIEZO1$^{\Delta EC}$ mouse.

(B) Mean ± SEM currents from experiments of the type exemplified in (A) (n = 6 recordings and N = 3 mice for WT and n = 8 and N = 3 for PIEZO1$^{\Delta EC}$).

(C and D) Representative recordings showing responses to −20 mmHg steps of 200 ms duration for endothelium from WT mice (C) and *Smpd3$^{fro/fro}$* (*fro/fro*) mice (D).

(E) Mean ± SEM data for the peak (maximum) inward current amplitude that occurred in experiments of the type shown in (C) and (D): WT recordings (n = 6 recordings, N = 3 mice) and *Smpd3$^{fro/fro}$* (*fro/fro*) recordings (n = 6, N = 3). ***p < 0.001.

(F) As for (E) but showing analysis of the current decay rate for WT (n = 6) and *Smpd3$^{fro/fro}$* (*fro/fro*) (n = 6) recordings.

(G) Mean ± SEM for experiments of the type shown in (C) and (D), showing the time for 20% decay of the current after reaching the peak: WT (n = 6 recordings, N = 3 mice) and *Smpd3$^{fro/fro}$* (*fro/fro*) (n = 6 recordings, N = 3 mice). ***p < 0.001 by Student's t test.

(H) Mean ± SEM data for experiments of the type shown in (C) and (D), showing the time to reach peak inward current after first applying the pressure step: WT (n = 6 recordings, N = 3 mice) and *Smpd3$^{fro/fro}$* (*fro/fro*) (n = 6 recordings, N = 3 mice).

amplitude (Figure S2). The data suggest that SMPD3 also prevents inactivation when the mechanical stimulus is a pressure pulse.

Ceramide Rescues the Membrane Potential Response to Flow

To test the roles of sphingomyelin and ceramide, we added them after SMPD3 had been inhibited by GW4869 to see if we could rescue the non-inactivating property of PIEZO1. We first recorded the membrane potential from endothelium. As expected, in the presence of GW4869, the flow response was transient (Figure 5A). When flow was repeated in the presence of 10 μM sphingomyelin, the response was again transient and indistinguishable from the first flow response in the absence of sphingomyelin, except for a slightly smaller peak amplitude (Figures 5B and 5C). In contrast, when the same experiment was repeated using 10 μM ceramide, there was significant rescue of the sustained response to flow (Figures 5D–5F). To investigate if the rescue was PIEZO1 dependent, we repeated the experiments with endothelium from

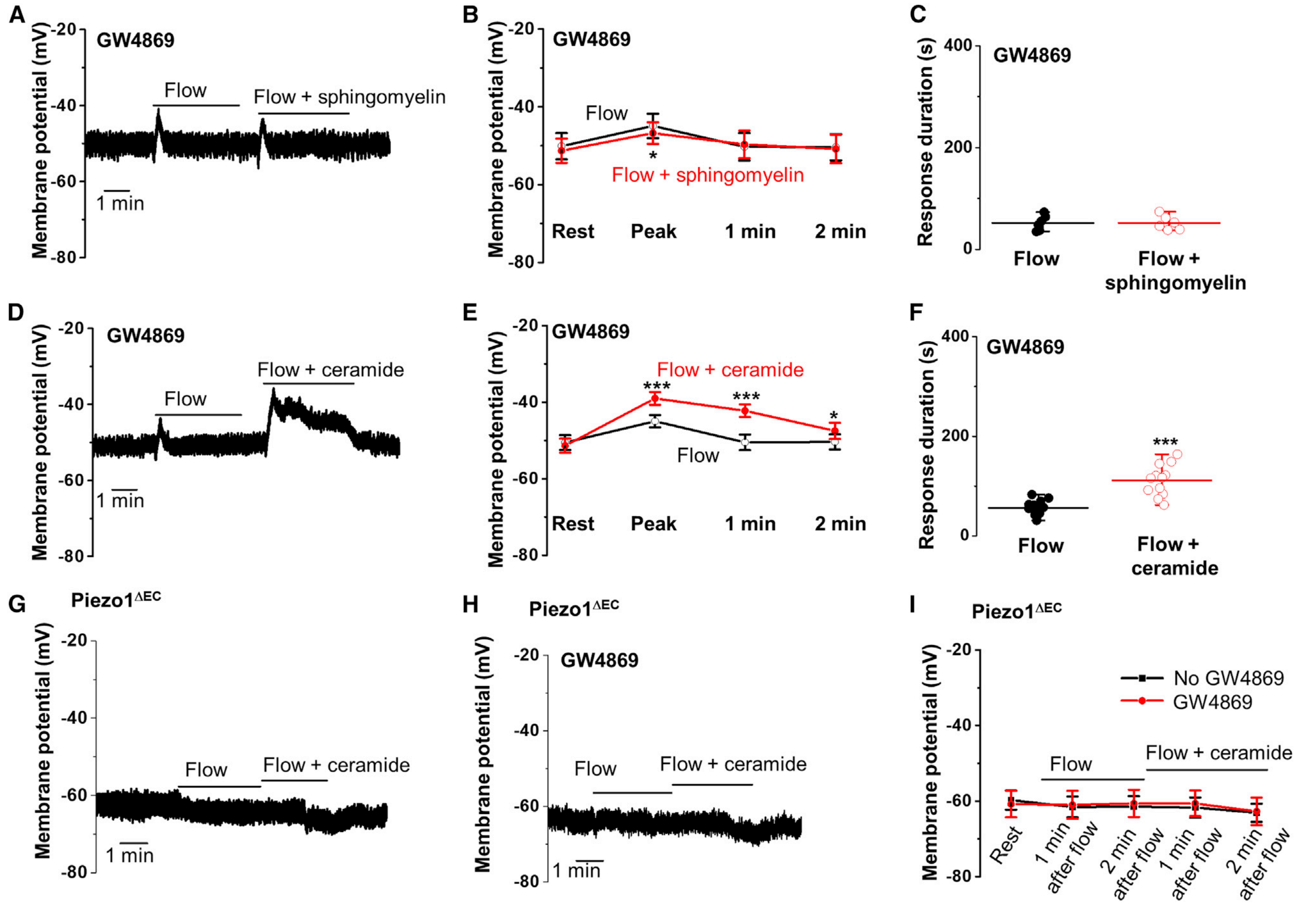

Figure 5. Ceramide Rescues Sustained Flow-Evoked Depolarization after Inhibition of Neutral Sphingomyelinase

All data relate to the membrane potential recorded in amphotericin whole-cell mode from freshly isolated endothelium of second-order mesenteric arteries.

(A) Representative recording showing responses to 20 μL s^{-1} fluid flow in the presence of 10 μM GW4869 in the absence and then presence of 10 μM sphingomyelin.

(B) Membrane potential mean ± SEM data for experiments of the type shown in (A).

(C) Response duration mean ± SEM data for experiments of the type shown in (A). Original raw data points are superimposed. (B and C) n = 6 recordings, N = 3 mice.

(D) Representative recording showing responses to 20 μL s^{-1} fluid flow in the presence of 10 μM GW4869 in the absence and then presence of 10 μM ceramide.

(E) Membrane potential mean ± SEM data for experiments of the type shown in (D).

(F) Response duration mean ± SEM data for experiments of the type shown in (D). Original raw data points are superimposed. (E and F) n = 12 recordings, N = 5 mice.

(G–I) Data from endothelium of PIEZO1$^{\Delta EC}$ mice. (G and H) Representative recordings showing responses to 20 μL s^{-1} fluid flow in the absence and then presence of 10 μM ceramide and in the absence (G) and presence (H) of 10 μM GW4869. (I) Membrane potential mean ± SEM data for experiments of the type exemplified in (G) and (H): n = 6 recordings and N = 3 mice for both groups.

PIEZO1$^{\Delta EC}$ mice. In these mice, there was no flow response and ceramide had no effect, whether GW4869 was present or not, suggesting that the flow and ceramide effects depended on PIEZO1 (Figures 5G–5I). Matched data for WT mice are shown for comparison in Figure S3. Therefore, the sustainability of cell depolarization was abolished by the inhibition of SMPD3 and recovered by acute application of ceramide. The immediate product of sphingomyelinase activity alongside ceramide is phosphoryl choline (PC), but application of 10 μM PC in the form 2-methacryloyloxyethyl PC had no effect (Figure S3). The data suggest that ceramide is the lipid that disables the PIEZO1 inactivation gate.

Ceramide Rescue Is Membrane Delimited

To further investigate the role of ceramide, we used outside-out patches for membrane-delimited studies and observation of the single-channel signature of PIEZO1 channels. Again, recordings were made in the presence of GW4869, which conferred a transient response to flow (Figure 6A; Figure S1). An addition of 10 μM sphingomyelin had no effect (Figures 6A–6C), whereas Gd^{3+}, the PIEZO1 channel inhibitor, suppressed the activity (Figure 6A). Amplitude histogram analysis showed the unitary current amplitude to be ~1.95 pA and thus the expected value for PIEZO1 channels (Figures 6A1 and 6A2). In contrast to the lack of an effect of sphingomyelin,

CellPress
OPEN ACCESS

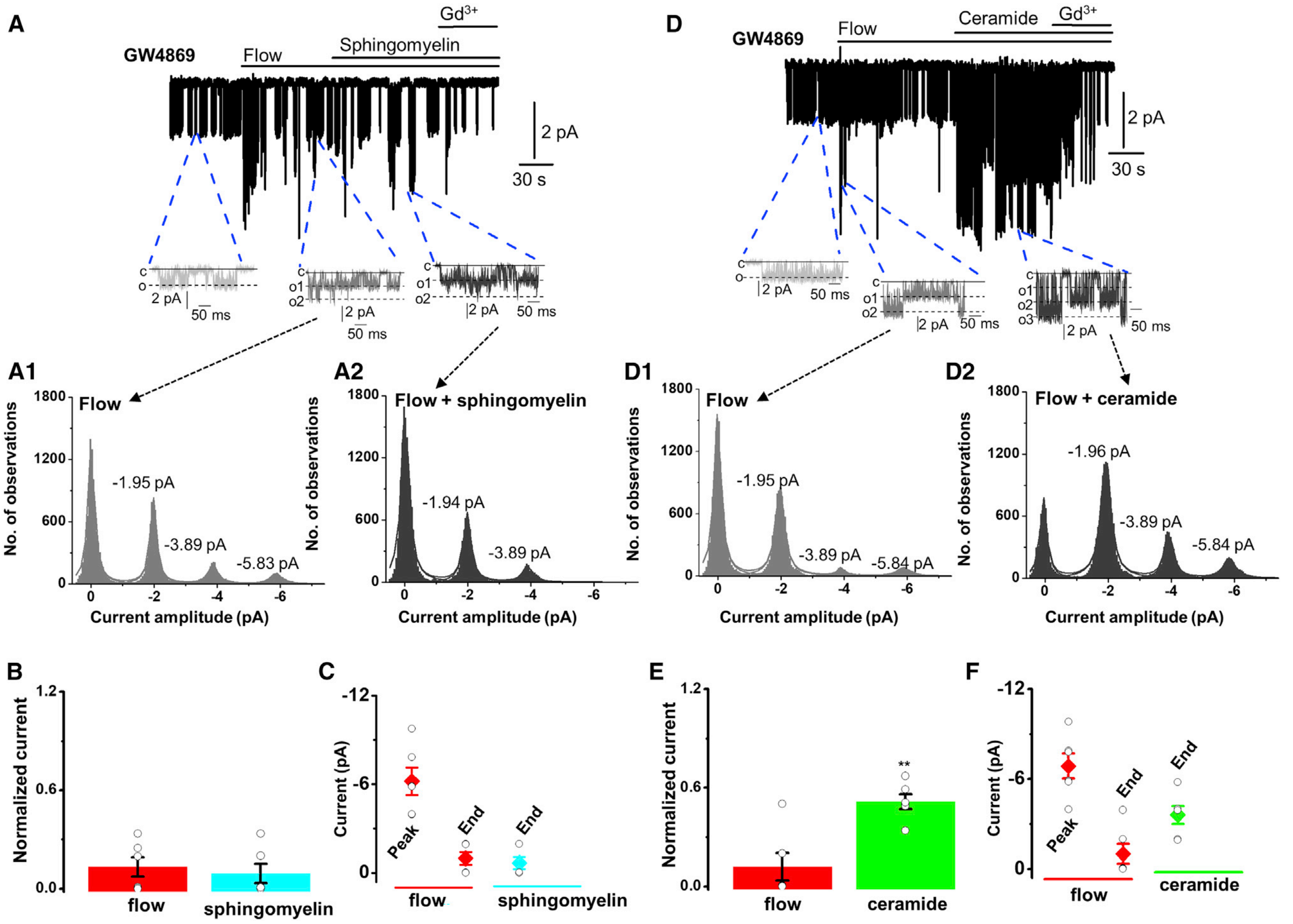

Figure 6. Ceramide Rescues Sustained Flow-Evoked Single-Channel Activity after Inhibition of Neutral Sphingomyelinase

All measurements were single-channel current recordings from outside-out patches excised from freshly isolated endothelium of second-order mesenteric arteries. Holding potential was −80 mV.

(A) Representative recording showing response to 20 μL s^{-1} fluid flow in the presence of 10 μM GW4869 in the absence and then presence of 10 μM sphingomyelin and then plus 10 μM Gd^{3+}. Three sections of the traces are shown below on the expanded timescale. (A1 and A2) Amplitude histograms for channel activity in flow and flow plus sphingomyelin, as shown in (A). Current amplitudes for peaks of the fitted distributions are indicated.

(B) For experiments of the type shown in (A), all data points and mean ± SEM for the channel currents at the end of recordings with the application of sphingomyelin (before the addition of Gd^{3+}) normalized to peak currents evoked by flow (n = 6 recordings, N = 3 mice).

(C) The values of peak current or the current at the end of recording for each patch shown in (A).

(D) Representative recording showing response to 20 μL s^{-1} fluid flow in the presence of 10 μM GW4869 in the absence and then presence of 10 μM ceramide and then plus 10 μM Gd^{3+}. Three sections of the traces are shown below on the expanded timescale. (D1 and D2) Amplitude histograms for channel activity in flow and flow plus ceramide, as shown in (D). Current amplitudes for peaks of the fitted distributions are indicated.

(E) For experiments of the type shown in (D), all data points and mean ± SEM for the channel currents at the end of recordings with the application of ceramide (before the addition of Gd^{3+}) normalized to peak currents evoked by flow (n = 6 recordings, N = 3 mice). **$p < 0.01$.

(F) The values of peak current or the current at the end of recording for each patch shown in (D).

ceramide strongly rescued channel activity after the initial transient response to flow in the presence of GW4869 (Figures 6D–6F). Gd^{3+} inhibited this channel activity, consistent with its mediation by PIEZO1 channels (Figure 6D). Moreover, the unitary current amplitude was the expected value for PIEZO1 channels and indistinguishable from that activated by flow alone (Figure 6D2 cf. Figure 6D1). As in membrane potential recordings, PC was ineffective (Figure S4). The data are consistent with ceramide disabling the inactivation gate by a membrane-delimited mechanism.

Ceramide Rescues the Non-inactivating Response to Pressure

As indicated above, GW4869 pre-treatment alone for 10 mins caused pressure-activated currents in cell-attached patches to become rapidly inactivating (Figures 7A, 7B, and 7C). We tested the acute application of lipids on PIEZO1 in cells pre-treated with GW4869. In the presence of ceramide, the inactivation was strongly and apparently selectively inhibited (Figures 7A, 7A1, 7A2, 7D, and 7G). In contrast, sphingomyelin and PC had no effect, failing to rescue the non-activating property (Figures 7B,

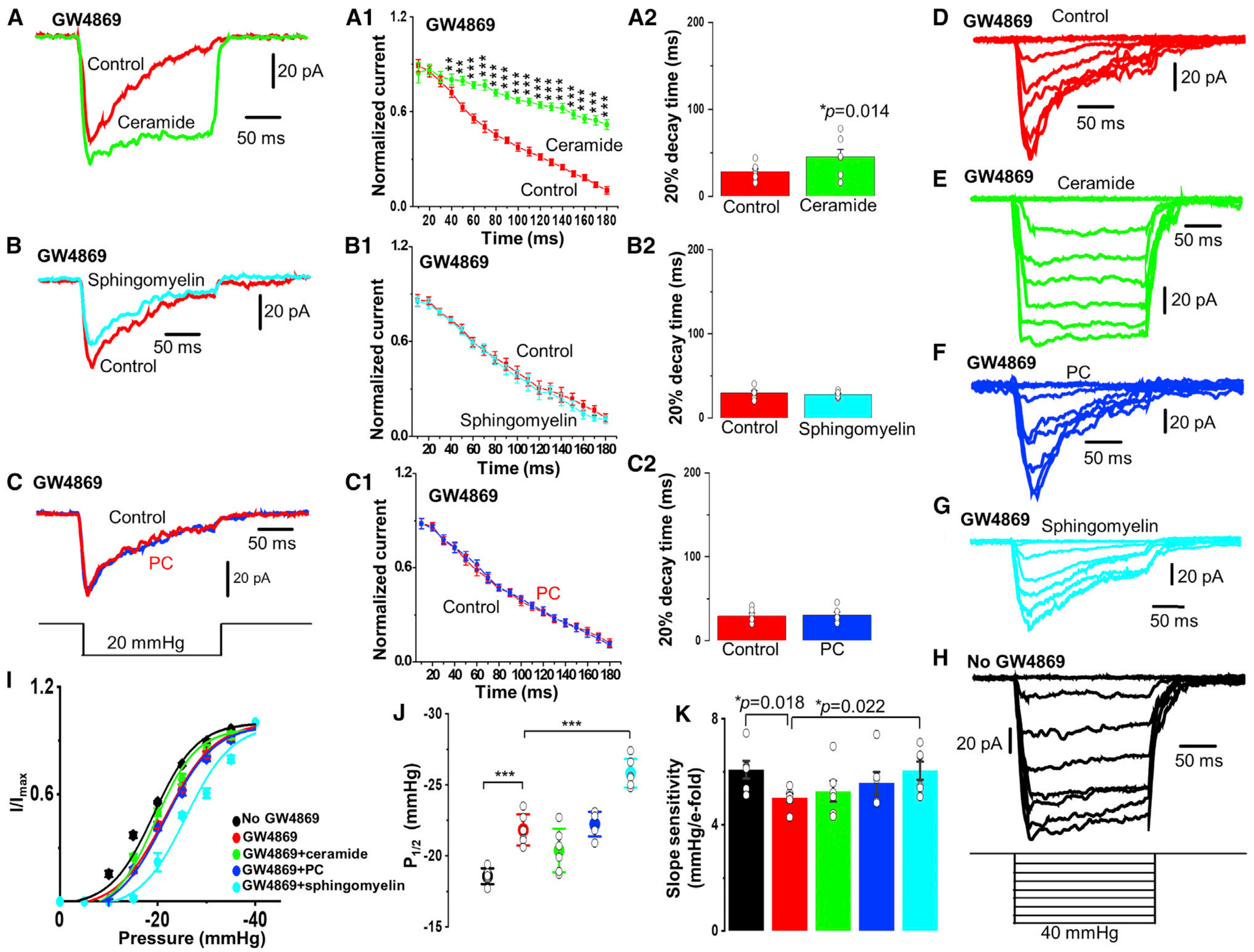

Figure 7. Ceramide and Sphingomyelin Have Distinct Effects

All measurements were made from cell-attached patches on freshly isolated endothelium of second-order mesenteric arteries of adult WT mice. The lipids were acutely applied in the experiments exemplified in (A)–(C), whereas cells were pre-treated with the lipids for 10 mins in the experiments exemplified in (D)–(H).

(A) Representative currents elicited by −20 mmHg pressure steps in the presence of 10 µM GW4869 and absence (control, red) and presence of 10 µM ceramide (green).

(B) Representative currents elicited by −20 mmHg pressure steps in the presence of 10 µM GW4869 and absence (control, red) and presence of 10 µM sphingomyelin (cyan).

(C) Representative currents elicited by −20 mmHg pressure steps in the presence of 10 µM GW4869 and absence (control, red) and presence of 10 µM PC (blue).

(A1 and A2) As for (A) but showing mean ± SEM data for the current decay rate and the time for 20% decay of the current after reaching the peak in the absence and presence of 10 µM ceramide (n = 7 recordings, N = 3 mice). *$p < 0.05$. **$p < 0.01$. ***$p < 0.001$. (B1 and B2) As for (B) but showing mean ± SEM data for the current decay rate and the time for 20% decay of the current after reaching the peak in the absence and presence of 10 µM sphingomyelin (n = 6 recordings, N = 3 mice). (C1 and C2) As for (C) but showing mean ± SEM data for the current decay rate and the time for 20% decay of the current after reaching the peak in the absence and presence of 10 µM PC (n = 6 recordings, N = 3 mice). **$p < 0.01$ and ***$p < 0.001$ by Student's t test.

(D–H) Representative currents elicited by increasing negative pressure steps from −5 to −40 mmHg, as illustrated schematically in the bottom panel. All recordings were in the presence of 10 µM GW4869 and the absence (D) or presence of 10 µM ceramide (E), 10 µM 2-methacryloyloxyethyl PC (PC) (F), or 10 µM sphingomyelin (G). (H) Recording in vehicle-control conditions.

(I) For experiments of the type exemplified in (D)–(H), mean ± SEM current amplitude normalized to the maximum at −40 mmHg plotted against the amplitude of the pressure step. Data are shown for vehicle control (black), in the presence of 10 µM GW4869 (red), in the presence of 10 µM GW4869 and 10 µM ceramide (green), in the presence of 10 µM GW4869 and 10 µM 2-methacryloyloxyethyl PC (PC; blue), and in the presence of 10 µM GW4869 and 10 µM sphingomyelin (cyan). Each dataset is for n = 6 recordings and N = 3 mice.

(J) Generated from the data shown in (I); mean ± SEM pressure for half-maximal activation ($P_{1/2}$). ***$p < 0.001$.

(K) Generated from the data shown in (I); mean ± SEM slope of the sigmoid fit (indicating force sensitivity). p = 0.018, p = 0.022, respectively, as indicated.

7B1, 7B2, 7C, 7C1, and 7C2). The data further support the conclusion that ceramide disables the inactivation gate.

Sphingomyelin Alters Pressure Sensitivity

Pressure sensitivity of PIEZO1 channels could also be influenced by membrane lipid composition (Douguet and Honoré, 2019). Therefore, we sought to determine whether SMPD3 has an additional effect on pressure sensitivity of native endothelial PIEZO1 channels. In control cell-attached patches, currents were progressively activated by pressure pulses of −5 mmHg or greater, reaching a maximum at about −40 mmHg (Figure 4B), which generated a saturating effect in most experimental groups. Unfortunately, patch recording was prone to disruption by pressure pulses greater than −40 mmHg in the cells pre-treated with GW4869 or additional lipids. A similar methodological challenge has also been reported in other type of cells when they were treated with molecules influencing lipid bilayers (Bae et al., 2011). Currents at all pressure steps were non-inactivating or slowly and partially inactivating (Figure 4A). In the presence of GW4869, all currents were inactivating (Figure 7D). In contrast, when ceramide was added along with GW4869, currents returned to non-activating behavior (Figure 7E). Sphingomyelin and PC had no obvious effects on inactivation (Figures 7F and 7G). The position of the current-pressure curve was right-shifted by the inhibitor of SMPD3 GW4869 (Figures 7I and 7J). Ceramide showed a tendency to reverse this effect (but the effect did not achieve statistical significance), whereas sphingomyelin moved the curve further to the right (Figures 7I and 7J). GW4869 caused the curve to have a shallower slope (suggesting lower pressure sensitivity) and this was reversed only by sphingomyelin (Figure 7K). The data suggest that SMPD3 also affects the position of the pressure curve and the sensitivity of the channels to pressure but that these effects are primarily mediated by sphingomyelin rather than ceramide.

DISCUSSION

Our findings build on previous work showing that fluid flow stimulates neutral sphingomyelinase and increases vascular ceramide (Czarny et al., 2003; Czarny and Schnitzer, 2004). We suggest that endothelial cells use this sphingomyelinase-based mechanism to alter the local lipid environment of PIEZO1 channels and thereby suppress inherent inactivation and alter force sensitivity, thus enabling physiologically important responses to mechanical stimuli such as fluid flow. We suggest that the specific sphingomyelinase subtype is SMPD3 and that the local lipid changes are increased ceramide (for suppressed inactivation) and reduced sphingomyelin (for altered force sensitivity).

SMPD3 is at the inner leaflet of the bilayer and interacts with calcineurin, a Ca^{2+}- and calmodulin-dependent serine/threonine protein phosphatase that activates SMPD3 by regulating its phosphorylation status (Airola and Hannun, 2013; Filosto et al., 2010). Because fluid-flow-evoked increases in ceramides have been detected within 1 min (Czarny et al., 2003), they could be fast enough to explain the sustained PIEZO1 activation by fluid flow in our membrane potential and single-channel recordings.

We suggest that ceramide generated by SMPD3 largely removes PIEZO1 inactivation when the PIEZO1 is in the native endothelium. Pre-treatment of these endothelial cells with ceramide for 10 mins or acute application of ceramide conferred similar non-inactivation properties on PIEZO1 in SMPD3-inhibited (GW4869-treated) endothelial cells. The effect caused by acute application of ceramide was slightly less obvious, but this could be due to the reduced delivery efficiency of ceramide when it is exogenously applied. Other lipids such as polyunsaturated fatty acids also inhibit PIEZO1 inactivation (Romero et al., 2019) and are likely to contribute to effects in the endothelium, depending on context.

We provide multiple lines of evidence for our claim that SMPD3 has the proposed role in regulating endothelial PIEZO1: pharmacology, gene modification, and lipid effects. The signals were PIEZO1 dependent because they were absent in PIEZO1$^{\Delta EC}$ mice. We also argue that the channels were PIEZO1 channels based on their distinctive unitary conductance of about 25 pS, which is the expected conductance for PIEZO1 channels and different from that of many other ion channels, including those that have been suggested to be activated by fluid flow (Coste et al., 2010; Li et al., 2014; Murthy et al., 2017; Rode et al., 2017). Moreover, the channels were robustly activated by mechanical force (a defining feature of PIEZO channels) and inhibited by Gd^{3+} (an expected but not unique feature of these channels). The similarity in the effects of neutral sphingomyelinase inhibitors and *Smpd3*$^{fro/fro}$ on endothelial PIEZO1 inactivation is particularly striking and argues in favor of a common mechanism, which we suggest to be SMPD3 (Figures 2, 3, and S5). A difference between the neutral sphingomyelinase inhibitor and *Smpd3*$^{fro/fro}$ effects was that *Smpd3*$^{fro/fro}$ mice showed a larger initial current in response to pressure pulse (Figure 4E). We do not know the explanation for this effect, but it may be due to compensation due to the developmental disruption of SMPD3 in the mice compared with the acute effects of the neutral sphingomyelinase inhibitors. We suggest that the currents in *Smpd3*$^{fro/fro}$ mice were indeed due to PIEZO1 channels because they had the expected unitary conductance, were activated by fluid flow, were inhibited by Gd^{3+}, and showed rapid inactivation of the type described for PIEZO1 in earlier studies (Coste et al., 2010; Zheng et al., 2019). The initial large current seen in voltage-clamp experiments contrasted with the small initial depolarization seen in constant current mode. This could be due to the integrated nature of the depolarization event, with other ion permeability mechanisms countering any overshoot depolarization.

In complementary studies, we observed that exogenous sphingomyelinase could modulate Ca^{2+} signals evoked by Yoda1, a chemical agonist of PIEZO1 (Figure S6). Such studies may be relevant to mechanical activation of the channels because Yoda1 is thought to sensitize the channels to mechanical force (Syeda et al., 2015). Intriguingly, the sphingomyelinase had the opposite effect on overexpressed PIEZO1 compared with native endothelial PIEZO1, suggesting that the standard methodological approach of characterizing PIEZO1 overexpressed in a host cell line may generate results that are not necessarily relevant to the physiological setting. There is emerging evidence for multiple complex interactions of PIEZO1 with various lipids (Chong et al., 2019; Romero et al., 2019), and so, the complement of lipids in the vicinity of each channel may determine which other lipids can act and then the type of effect of each lipid. Intriguingly, the previous reports of effects of exogenous sphingomyelinase on gating properties of

voltage-gated ion channels, such as the slowing of inactivation in voltage-gated Na^+ channels, also depended on the host cell type (Combs et al., 2013).

It will be interesting to investigate if the SMPD3-dependent escape of PIEZO1 from inactivation and regulation of its force sensitivity are important in other, non-endothelial cell types for which rapid inactivation is also likely to be incompatible with function. An important cell type for investigation will be the red blood cell for which mutations in *PIEZO1* lead to dehydrated hereditary stomatocytosis (Andolfo et al., 2013) and the PIEZO1 channels are non-inactivating or only slowly inactivating (Evans et al., 2020). Osteoblasts also exhibit non-inactivating PIEZO1 activity (Sun et al., 2019), and it may not be a coincidence that *PIEZO1*- and *Smpd3*-disrupted mice both show abnormal bone formation (Aubin et al., 2005; Sun et al., 2019). Slow or little inactivation has also been described in other cell types (Beech and Kalli, 2019) such as embryonic stem cells (for which a role of PIEZO1 in the relatively slow process of cell proliferation has been suggested) (Del Mármol et al., 2018) and other cells including cardiac fibroblasts (Blythe et al., 2019), smooth muscle cells (Retailleau et al.,2015), and renal epithelial cells (Peyronnet et al., 2013).

Based on the available evidence, we cannot say whether shear stress acts by first stimulating SMPD3 to release PIEZO1 from inactivation or whether SMPD3 is already sufficiently active to enable a non-inactivating PIEZO1 that is then the first protein activated by shear stress. Moreover, it is important to emphasize that we do not infer that the sphingolipid mechanism identified here is specific to PIEZO1 because sphingolipids (in particular the ceramides and their associated cascade of lipid products) are well-established as having multiple complex effects on cells (Hannun and Obeid, 2018; Pavoine and Pecker, 2009). Stimulation of sphingomyelinase by shear stress (Czarny et al., 2003; Czarny and Schnitzer, 2004) is likely to have many downstream implications. Furthermore, we do not argue that PIEZO1 is the only shear stress sensor or that it is necessarily the first protein to be activated by shear stress among the many candidate sensors so far described (Baratchi et al., 2017). Future studies will hopefully unravel the complexities of the overall shear stress-sensing system, including the order in which the various events occur and the anatomical sites and contexts in which the different factors prevail.

Our study does not reveal the molecular mechanisms by which ceramide or sphingomyelin modulate the function of PIEZO1 channels. To explore potential ideas, we used molecular dynamics simulations based on the available structural data for mouse PIEZO1 (Figure S7). Although these data lack information for N-terminal regions, they include high-resolution data for the central pore region and parts of the blades, which participate in force sensing. Ceramide lacks the head group of sphingomyelin, so replacing sphingomyelin with ceramide in a membrane is likely to change some properties of the membrane such as curvature. Changes in membrane curvature can be important, as PIEZO1 channels alter the membrane structure to create an inwardly directed dome, which has been suggested to be important in PIEZO1 activation (Guo and MacKinnon, 2017; Lin et al., 2019). Although it is possible to see spontaneous membrane curvature in simulations (Hossein and Deserno, 2020), theoretical and experimental studies have shown that PIEZO1 in a membrane does indeed create an inward-facing dome (Guo and MacKinnon, 2017). The depth of the previously suggested dome was ~6 nm, which is similar to our simulations (Figure S7) and other simulations in a 1-palmitoyl-2-oleyl-phosphtidylcholine (POPC) membrane (Botello-Smith et al., 2019). This model suggests that sphingomyelinase activity reduces the depth of the dome by generating ceramide at the expense of sphingomyelin. As with any technique, limitations of simulations exist. However, they enable otherwise unachievable insight into dynamic molecular mechanisms, and studies with realistic membranes (as we performed) are becoming the norm in such simulations, with increasing evidence that they can predict properties of native membranes (Marrink et al., 2019). Nevertheless, we recognize that the changes in the dome depth were relatively modest and the functional significance of such changes is not yet known. It would be premature to rule out other mechanisms that may include, for example, altered membrane thickness (because ceramides lack the headgroup of sphingomyelin), increased shear membrane viscosity caused by ceramide (Catapano et al., 2017), and direct lipid interactions with PIEZO1 amino acids. Multiple mechanisms are indicated because the effects of sphingomyelin and ceramide were not simple opposites but rather distinct effects. Although previous work (Wu et al., 2017) suggested that membrane tension has little role in inactivation kinetics of PIEZO1, this study was carried out in PIEZO1-overexpressing HEK293 cells, which our data suggest may not necessarily reproduce properties of PIEZO1 in native endothelial cells.

In conclusion, we suggest that a sphingomyelinase (SMPD3) suppresses PIEZO1 inactivation by catalyzing the production of ceramides that favor the channel open state over closed states such as the inactivated state. Although we support this conclusion through potentially non-physiological experiments in which we added exogenous lipids to the endothelium, we argue that the mechanism is likely to be physiologically important because genetic disruption of SMPD3, which physiologically regulates these lipids in their native membrane context, remarkably switched the channels to the rapidly inactivating behavior seen often in overexpression systems and natively in some other cell types. The latter effect of SMPD3's disruption on PIEZO1 biophysics is unlikely to be explained by a grossly disturbed phenotype of the *Smpd3*$^{fro/fro}$ mouse because we could easily reproduce it if we acutely inhibited the enzyme pharmacologically in WT endothelium. We suggest therefore that sphingomyelinase activity is a major determinant of native PIEZO gating. This effect may have broad cell and tissue consequences beyond those described here for the mesenteric endothelium and extend to other ion channel types that were previously shown to be modulated by exogenous sphingomyelinase and perhaps other ion channels and other membrane proteins that have not yet been investigated in relation to sphingomyelinase.

STAR★METHODS

Detailed methods are provided in the online version of this paper and include the following:

- KEY RESOURCES TABLE
- RESOURCE AVAILABILITY
  - Lead Contact
  - Materials Availability

- ○ Data and Code Availability
- EXPERIMENTAL MODEL AND SUBJECT DETAILS
  - ○ Animals
- METHOD DETAILS
  - ○ Isolation of Endothelium from Mesenteric Artery
  - ○ Patch-clamp Electrophysiology
  - ○ Culture and Transfection of Human Umbilical Vein Endothelial Cells (HUVECs)
  - ○ PIEZO1 Tetracycline Inducible HEK293 Cell Line
  - ○ Intracellular Ca^{2+} Measurement
  - ○ PIEZO1 Channel Modeling
  - ○ Coarse-Grained Simulations
  - ○ Trajectory Analyses and Molecular Graphics
- QUANTIFICATION AND STATISTICAL ANALYSIS

SUPPLEMENTAL INFORMATION

Supplemental Information can be found online at https://doi.org/10.1016/j.celrep.2020.108225.

ACKNOWLEDGMENTS

The research was supported by a British Heart Foundation Intermediate Research Fellowship to J.S.; Wellcome Trust Investigator and British Heart Foundation programme grants to D.J.B.; an Academy of Medical Sciences and Wellcome Trust Springboard Award to A.C.K.; and INSERM funding to M.R., A.N.S., and N.A. A.J.H. was supported by a BBSRC PhD Studentship and J.C. by a MRC Clinical Research Training fellowship.

AUTHOR CONTRIBUTIONS

J.S. performed and analyzed patch-clamp recordings and isolated endothelial cells. A.J.H. performed intracellular Ca^{2+} measurements. D.D.V. and A.C.K. generated the PIEZO1 molecular model. D.D.V. performed and analyzed the simulations. J.C. wrote the script for analyzing dome depth. L.L., T.S.F., M.R., A.N.S., and N.A. prepared genetically modified mice. D.J.B., J.S., and A.C.K. raised funds for the project, generated ideas, interpreted data, and co-wrote the paper. D.J.B. and J.S. organized the project.

DECLARATION OF INTERESTS

The authors declare no competing financial interests.

Received: April 28, 2020
Revised: July 30, 2020
Accepted: September 11, 2020
Published: October 6, 2020

SUPPORTING CITATIONS

The following reference appears in the Supplemental Information: Takamura et al., 1990.

REFERENCES

Abraham, M.J., Murtola, T., Schulz, R., Páll, S., Smith, J.C., Hess, B., and Lindahl, E. (2015). GROMACS: High performance molecular simulations through multi-level parallelism from laptops to supercomputers. SoftwareX *1-2*, 19–25.

Airola, M.V., and Hannun, Y.A. (2013). Sphingolipid metabolism and neutral sphingomyelinases. Handb. Exp. Pharmacol. *215*, 57–76.

Albarrán-Juárez, J., Iring, A., Wang, S., Joseph, S., Grimm, M., Strilic, B., Wettschureck, N., Althoff, T.F., and Offermanns, S. (2018). Piezo1 and G_q/G_{11} promote endothelial inflammation depending on flow pattern and integrin activation. J. Exp. Med. *215*, 2655–2672.

Andolfo, I., Alper, S.L., De Franceschi, L., Auriemma, C., Russo, R., De Falco, L., Vallefuoco, F., Esposito, M.R., Vandorpe, D.H., Shmukler, B.E., et al. (2013). Multiple clinical forms of dehydrated hereditary stomatocytosis arise from mutations in PIEZO1. Blood *121*, 3925–3935, S1–S12.

Aubin, I., Adams, C.P., Opsahl, S., Septier, D., Bishop, C.E., Auge, N., Salvayre, R., Negre-Salvayre, A., Goldberg, M., Guénet, J.L., and Poirier, C. (2005). A deletion in the gene encoding sphingomyelin phosphodiesterase 3 (Smpd3) results in osteogenesis and dentinogenesis imperfecta in the mouse. Nat. Genet. *37*, 803–805.

Bae, C., Sachs, F., and Gottlieb, P.A. (2011). The mechanosensitive ion channel Piezo1 is inhibited by the peptide GsMTx4. Biochemistry *50*, 6295–6300.

Baratchi, S., Khoshmanesh, K., Woodman, O.L., Potocnik, S., Peter, K., and McIntyre, P. (2017). Molecular Sensors of Blood Flow in Endothelial Cells. Trends Mol. Med. *23*, 850–868.

Beech, D.J., and Kalli, A.C. (2019). Force Sensing by Piezo Channels in Cardiovascular Health and Disease. Arterioscler. Thromb. Vasc. Biol. *39*, 2228–2239.

Berendsen, H.J.C., Postma, J.P.M., van Gunsteren, W.F., DiNola, A., and Haak, J.R. (1984). Molecular dynamics with coupling to an external bath. J. Chem. Phys. *81*, 3684–3690.

Blythe, N.M., Muraki, K., Ludlow, M.J., Stylianidis, V., Gilbert, H.T.J., Evans, E.L., Cuthbertson, K., Foster, R., Swift, J., Li, J., et al. (2019). Mechanically activated Piezo1 channels of cardiac fibroblasts stimulate p38 mitogen-activated protein kinase activity and interleukin-6 secretion. J. Biol. Chem. *294*, 17395–17408.

Botello-Smith, W.M., Jiang, W., Zhang, H., Ozkan, A.D., Lin, Y.C., Pham, C.N., Lacroix, J.J., and Luo, Y. (2019). A mechanism for the activation of the mechanosensitive Piezo1 channel by the small molecule Yoda1. Nat. Commun. *10*, 4503.

Bussi, G., Donadio, D., and Parrinello, M. (2007). Canonical sampling through velocity rescaling. J. Chem. Phys. *126*, 014101.

Catapano, E.R., Natale, P., Monroy, F., and López-Montero, I. (2017). The enzymatic sphingomyelin to ceramide conversion increases the shear membrane viscosity at the air-water interface. Adv. Colloid Interface Sci. *247*, 555–560.

Chiu, J.J., and Chien, S. (2011). Effects of disturbed flow on vascular endothelium: pathophysiological basis and clinical perspectives. Physiol. Rev. *91*, 327–387.

Chong, J., De Vecchis, D., Hyman, A.J., Povstyan, O.V., Shi, J., Beech, D.J., and Kalli, A.C. (2019). Computational reconstruction of the complete Piezo1 structure reveals a unique footprint and specific lipid interactions. bioRxiv. https://doi.org/10.1101/783753.

Combs, D.J., Shin, H.G., Xu, Y., Ramu, Y., and Lu, Z. (2013). Tuning voltage-gated channel activity and cellular excitability with a sphingomyelinase. J. Gen. Physiol. *142*, 367–380.

Corradi, V., Sejdiu, B.I., Mesa-Galloso, H., Abdizadeh, H., Noskov, S.Y., Marrink, S.J., and Tieleman, D.P. (2019). Emerging Diversity in Lipid-Protein Interactions. Chem. Rev. *119*, 5775–5848.

Coste, B., Mathur, J., Schmidt, M., Earley, T.J., Ranade, S., Petrus, M.J., Dubin, A.E., and Patapoutian, A. (2010). Piezo1 and Piezo2 are essential components of distinct mechanically activated cation channels. Science *330*, 55–60.

Czarny, M., and Schnitzer, J.E. (2004). Neutral sphingomyelinase inhibitor scyphostatin prevents and ceramide mimics mechanotransduction in vascular endothelium. Am. J. Physiol. Heart Circ. Physiol. *287*, H1344–H1352.

Czarny, M., Liu, J., Oh, P., and Schnitzer, J.E. (2003). Transient mechanoactivation of neutral sphingomyelinase in caveolae to generate ceramide. J. Biol. Chem. *278*, 4424–4430.

de Jong, D.H., Singh, G., Bennett, W.F., Arnarez, C., Wassenaar, T.A., Schäfer, L.V., Periole, X., Tieleman, D.P., and Marrink, S.J. (2013). Improved Parameters for the Martini Coarse-Grained Protein Force Field. J. Chem. Theory Comput. *9*, 687–697.

De Vecchis, D., Reithmeier, R.A.F., and Kalli, A.C. (2019). Molecular Simulations of Intact Anion Exchanger 1 Reveal Specific Domain and Lipid Interactions. Biophys. J. *117*, 1364–1379.

Del Mármol, J.I., Touhara, K.K., Croft, G., and MacKinnon, R. (2018). Piezo1 forms a slowly-inactivating mechanosensory channel in mouse embryonic stem cells. eLife *7*, e33149.

Douguet, D., and Honoré, E. (2019). Mammalian mechanoelectrical transduction: structure and function of force-gated ion channels. Cell *179*, 340–354.

Douguet, D., Patel, A., Xu, A., Vanhoutte, P.M., and Honoré, E. (2019). Piezo Ion Channels in Cardiovascular Mechanobiology. Trends Pharmacol. Sci. *40*, 956–970.

Evans, E.L., Cuthbertson, K., Endesh, N., Rode, B., Blythe, N.M., Hyman, A.J., Hall, S.J., Gaunt, H.J., Ludlow, M.J., Foster, R., and Beech, D.J. (2018). Yoda1 analogue (Dooku1) which antagonizes Yoda1-evoked activation of Piezo1 and aortic relaxation. Br. J. Pharmacol. *175*, 1744–1759.

Evans, E.L., Povstyan, O.V., De Vecchis, D., Macrae, F., Lichtenstein, L., Futers, T.S., Parsonage, G., Humphreys, N., Adamson, A., Kalli, A.C., et al. (2020). RBCs prevent rapid PIEZO1 inactivation and expose slow deactivation as a mechanism of dehydrated hereditary stomatocytosis. Blood *136*, 140–144.

Filosto, S., Fry, W., Knowlton, A.A., and Goldkorn, T. (2010). Neutral sphingomyelinase 2 (nSMase2) is a phosphoprotein regulated by calcineurin (PP2B). J. Biol. Chem. *285*, 10213–10222.

Fiser, A., and Sali, A. (2003). Modeller: generation and refinement of homology-based protein structure models. Methods Enzymol. *374*, 461–491.

Fotiou, E., Martin-Almedina, S., Simpson, M.A., Lin, S., Gordon, K., Brice, G., Atton, G., Jeffery, I., Rees, D.C., Mignot, C., et al. (2015). Novel mutations in PIEZO1 cause an autosomal recessive generalized lymphatic dysplasia with non-immune hydrops fetalis. Nat. Commun. *6*, 8085.

Friedrich, E.E., Hong, Z., Xiong, S., Zhong, M., Di, A., Rehman, J., Komarova, Y.A., and Malik, A.B. (2019). Endothelial cell Piezo1 mediates pressure-induced lung vascular hyperpermeability via disruption of adherens junctions. Proc. Natl. Acad. Sci. USA *116*, 12980–12985.

Guo, Y.R., and MacKinnon, R. (2017). Structure-based membrane dome mechanism for Piezo mechanosensitivity. eLife *6*, e33660.

Hannun, Y.A., and Obeid, L.M. (2018). Sphingolipids and their metabolism in physiology and disease. Nat. Rev. Mol. Cell Biol. *19*, 175–191.

Honoré, E., Martins, J.R., Penton, D., Patel, A., and Demolombe, S. (2015). The Piezo Mechanosensitive Ion Channels: May the Force Be with You!. Rev. Physiol. Biochem. Pharmacol. *169*, 25–41.

Hossein, A., and Deserno, M. (2020). Spontaneous Curvature, Differential Stress, and Bending Modulus of Asymmetric Lipid Membranes. Biophys. J. *118*, 624–642.

Humphrey, W., Dalke, A., and Schulten, K. (1996). VMD: visual molecular dynamics. J. Mol. Graph. *14*, 33–38, 27–38.

Kornhuber, J., Tripal, P., Reichel, M., Terfloth, L., Bleich, S., Wiltfang, J., and Gulbins, E. (2008). Identification of new functional inhibitors of acid sphingomyelinase using a structure-property-activity relation model. J. Med. Chem. *51*, 219–237.

Kumar, A., Henry, R.J., Stoica, B.A., Loane, D.J., Abulwerdi, G., Bhat, S.A., and Faden, A.I. (2019). Neutral Sphingomyelinase Inhibition Alleviates LPS-Induced Microglia Activation and Neuroinflammation after Experimental Traumatic Brain Injury. J. Pharmacol. Exp. Ther. *368*, 338–352.

Lallemand, T., Rouahi, M., Swiader, A., Grazide, M.H., Geoffre, N., Alayrac, P., Recazens, E., Coste, A., Salvayre, R., Nègre-Salvayre, A., and Augé, N. (2018). nSMase2 (Type 2-Neutral Sphingomyelinase) Deficiency or Inhibition by GW4869 Reduces Inflammation and Atherosclerosis in Apoe$^{-/-}$ Mice. Arterioscler. Thromb. Vasc. Biol. *38*, 1479–1492.

Li, J., Hou, B., Tumova, S., Muraki, K., Bruns, A., Ludlow, M.J., Sedo, A., Hyman, A.J., McKeown, L., Young, R.S., et al. (2014). Piezo1 integration of vascular architecture with physiological force. Nature *515*, 279–282.

Lin, Y.C., Guo, Y.R., Miyagi, A., Levring, J., MacKinnon, R., and Scheuring, S. (2019). Force-induced conformational changes in PIEZO1. Nature *573*, 230–234.

Luberto, C., Hassler, D.F., Signorelli, P., Okamoto, Y., Sawai, H., Boros, E., Hazen-Martin, D.J., Obeid, L.M., Hannun, Y.A., and Smith, G.K. (2002). Inhibition of tumor necrosis factor-induced cell death in MCF7 by a novel inhibitor of neutral sphingomyelinase. J. Biol. Chem. *277*, 41128–41139.

Mammoto, T., and Ingber, D.E. (2010). Mechanical control of tissue and organ development. Development *137*, 1407–1420.

Marrink, S.J., Risselada, H.J., Yefimov, S., Tieleman, D.P., and de Vries, A.H. (2007). The MARTINI force field: coarse grained model for biomolecular simulations. J. Phys. Chem. B *111*, 7812–7824.

Marrink, S.J., Corradi, V., Souza, P.C.T., Ingólfsson, H.I., Tieleman, D.P., and Sansom, M.S.P. (2019). Computational Modeling of Realistic Cell Membranes. Chem. Rev. *119*, 6184–6226.

Murthy, S.E., Dubin, A.E., and Patapoutian, A. (2017). Piezos thrive under pressure: mechanically activated ion channels in health and disease. Nat. Rev. Mol. Cell Biol. *18*, 771–783.

Parrinello, M.R.A. (1981). Polymorphic transitions in single crystals: A new molecular dynamics method. J. Appl. Phys. *52*, 7182–7190.

Pavoine, C., and Pecker, F. (2009). Sphingomyelinases: their regulation and roles in cardiovascular pathophysiology. Cardiovasc. Res. *82*, 175–183.

Peyronnet, R., Martins, J.R., Duprat, F., Demolombe, S., Arhatte, M., Jodar, M., Tauc, M., Duranton, C., Paulais, M., Teulon, J., et al. (2013). Piezo1-dependent stretch-activated channels are inhibited by Polycystin-2 in renal tubular epithelial cells. EMBO Rep. *14*, 1143–1148.

Retailleau, K., Duprat, F., Arhatte, M., Ranade, S.S., Peyronnet, R., Martins, J.R., Jodar, M., Moro, C., Offermanns, S., Feng, Y., et al. (2015). Piezo1 in smooth muscle cells is involved in hypertension-dependent arterial remodeling. Cell Rep. *13*, 1161–1171.

Rode, B., Shi, J., Endesh, N., Drinkhill, M.J., Webster, P.J., Lotteau, S.J., Bailey, M.A., Yuldasheva, N.Y., Ludlow, M.J., Cubbon, R.M., et al. (2017). Piezo1 channels sense whole body physical activity to reset cardiovascular homeostasis and enhance performance. Nat. Commun. *8*, 350.

Romero, L.O., Massey, A.E., Mata-Daboin, A.D., Sierra-Valdez, F.J., Chauhan, S.C., Cordero-Morales, J.F., and Vásquez, V. (2019). Dietary fatty acids fine-tune Piezo1 mechanical response. Nat. Commun. *10*, 1200.

Sali, A., and Blundell, T.L. (1993). Comparative protein modelling by satisfaction of spatial restraints. J. Mol. Biol. *234*, 779–815.

Shen, M.Y., and Sali, A. (2006). Statistical potential for assessment and prediction of protein structures. Protein Sci. *15*, 2507–2524.

Sun, W., Chi, S., Li, Y., Ling, S., Tan, Y., Xu, Y., Jiang, F., Li, J., Liu, C., Zhong, G., et al. (2019). The mechanosensitive Piezo1 channel is required for bone formation. eLife *8*, e47454.

Syeda, R., Xu, J., Dubin, A.E., Coste, B., Mathur, J., Huynh, T., Matzen, J., Lao, J., Tully, D.C., Engels, I.H., et al. (2015). Chemical activation of the mechanotransduction channel Piezo1. eLife *4*, e07369.

Takamura, H., Kasai, H., Arita, H., and Kito, M. (1990). Phospholipid molecular species in human umbilical artery and vein endothelial cells. J. Lipid Res. *31*, 709–717.

Van Der Spoel, D., Lindahl, E., Hess, B., Groenhof, G., Mark, A.E., and Berendsen, H.J.C. (2005). GROMACS: fast, flexible, and free. J. Comput. Chem. *26*, 1701–1718.

Wassenaar, T.A., Ingólfsson, H.I., Böckmann, R.A., Tieleman, D.P., and Marrink, S.J. (2015). Computational Lipidomics with insane: A Versatile Tool for Generating Custom Membranes for Molecular Simulations. J. Chem. Theory Comput. *11*, 2144–2155.

Wu, J., Young, M., Lewis, A.H., Martfeld, A.N., Kalmeta, B., and Grandl, J. (2017). Inactivation of Mechanically Activated Piezo1 Ion Channels Is Determined by the C-Terminal Extracellular Domain and the Inner Pore Helix. Cell Rep. *21*, 2357–2366.

Zheng, W., Gracheva, E.O., and Bagriantsev, S.N. (2019). A hydrophobic gate in the inner pore helix is the major determinant of inactivation in mechanosensitive Piezo channels. eLife *8*, e44003.

STAR★METHODS

KEY RESOURCES TABLE

REAGENT or RESOURCE	SOURCE	IDENTIFIER
Chemicals, Peptides, and Recombinant Proteins		
fura-2AM	Molecular Probes TM	Catalog #: 65-0858-39
Desipramine	Tocris	Catalog #: D3900
GW4869	Sigma-Aldrich	Catalog #: 567715
Altenusin	Sigma-Aldrich	Catalog #: SML2193
Sphingomyelin	Sigma-Aldrich	Catalog #: S0756
Ceramide	Sigma-Aldrich	Catalog #: 860516P
2-methacryloyloxyethyl phosphorylcholine	Sigma-Aldrich	Catalog #:730114
Collagenase Type IA	Sigma-Aldrich	Catalog #: C9891
Sphingomyelinase	Sigma-Aldrich	Catalog #:S9396
Yoda1	Tocris	Catalog #:5586
Experimental Models: Cell Lines		
Human Umbilical Vein Endothelial Cells (HUVEC)	Lonza	Catalog #: CC-2935
HEK T-REx cells	Evans et al., 2018	N/A
Experimental Models: Organisms/Strains		
Mouse: C57BL/6 strain males for PIEZO1 WT and PIEZO1$^{\Delta EC}$	Rode et al., 2017	N/A
Mouse: 129/Sv strain males for wild-type or *Smpd3*$^{fro/fro}$	Aubin et al., 2005	N/A
Oligonucleotides		
siRNA targeting sequence: PIEZO1: GCCUCGUGGUCUACAAGAUtt	Li et al., 2014	N/A
Software and Algorithms		
Softmax Pro software v5.4.5	Molecular Devices	https://www.moleculardevices.com/products/microplate-readers
GROMACS 5.0.7	Van Der Spoel et al., 2005	http://www.gromacs.org
MODELER (v 9.19)	Fiser and Sali, 2003	https://salilab.org/modeller/
Martini 2.2 force field	Marrink et al., 2007	http://www.cgmartini.nl/index.php
pCLAMP 10.6 software	Molecular Devices	https://www.moleculardevices.com/products/axon-patch-clamp-system
VMD 1.9	Humphrey et al., 1996	http://www.ks.uiuc.edu/Research/vmd/
Grace	Grace	https://plasma-gate.weizmann.ac.il/Grace/
Origin Pro	OriginLab	https://www.originlab.com/

RESOURCE AVAILABILITY

Lead Contact
Further information and requests for resources and reagents should be directed to and will be fulfilled by the Lead Contact, Jian Shi (j.shi1@leeds.ac.uk).

Materials Availability
This study did not generate new unique reagents.

Data and Code Availability
All datasets generated or analyzed during this study are included in the published article. All data are available from the Lead Contact upon reasonable request.

EXPERIMENTAL MODEL AND SUBJECT DETAILS

Animals

All animal use was authorized by the University of Leeds Animal Ethics Committee and The Home Office, UK. All animals were maintained in GM500 individually ventilated cages (Animal Care Systems) at 21°C 50%–70% humidity, light/dark cycle 12/12 hr on RM1 diet (SpecialDiet Services, Witham, UK) *ad libitum* and bedding of Pure'o Cell (Datesand, Manchester, UK). Genotypes were determined using real-time PCR with specific probes designed for each gene (Transnetyx, Cordova, TN). Male wild-type or *Smpd3*$^{fro/fro}$ mice (Aubin et al., 2005) on 129/Sv background were 12-13 weeks old at the time of experiments. PIEZO1$^{\Delta EC}$ have been previously described and were used similarly (Rode et al., 2017). Otherwise, all mice were C57BL/6 males aged 10-14 weeks.

METHOD DETAILS

Isolation of Endothelium from Mesenteric Artery

Endothelium was freshly isolated from second-order branches of mouse mesenteric arteries as described previously (Rode et al., 2017). Briefly, dissected second-order mesenteric arteries were enzymatically digested in dissociation solution (126 mM NaCl, 6 mM KCl, 10 mM Glucose, 11 mM HEPES, 1.2 mM $MgCl_2$, 0.05 mM $CaCl_2$, with pH titrated to 7.2) containing 1 mg.mL^{-1} collagenase Type IA (Sigma-Aldrich, Dorset, UK) for 14 min at 37°C and then triturated gently to release endothelium on a glass coverslips for recordings on the same day.

Patch-clamp Electrophysiology

Recordings were made at room temperature using an Axopatch-200B amplifier equipped with a Digidata 1550A and pCLAMP 10.6 software (Molecular Devices, Sunnyvale, CA, USA). Endothelium was in a standard bath solution containing (mM) 135 NaCl, 4 KCl, 2 $CaCl_2$, 1 $MgCl_2$, 10 glucose and 10 HEPES (titrated to pH 7.4 using NaOH). For membrane potential recordings in zero current mode, heat-polished patch pipettes with tip resistances between 3 and 5 MΩ were used and contained amphotericin B (Sigma-Aldrich) as the perforating agent, added to a pipette solution containing (mM) 145 KCl, 1 $MgCl_2$, 0.5 EGTA and 10 HEPES (titrated to pH 7.2 using KOH). Outside-out and cell-attached membrane patch recordings were made using the same equipment but in voltage-clamp mode. The tip resistances of recording pipettes for cell-attached recordings were between 3 and 5 MΩ, while for outside-out recordings they were between 12 and 15 MΩ. Currents were sampled at 20 kHz and filtered at 2 kHz. For cell-attached recordings, the extracellular (bath) solution contained (mM) 140 KCl, 1 $MgCl_2$, 10 glucose and 10 HEPES (titrated to pH 7.3 using KOH), while the patch pipette contained (mM) 130 NaCl, 5 KCl, 1 $CaCl_2$, 1 $MgCl_2$, 10 Tetraethylammonium.Cl and 10 HEPES (titrated to pH 7.3 using NaOH). For outside-out recordings, the external solution was standard bath solution and the patch pipette contained (mM) 145 KCl, 1 $MgCl_2$, 0.5 EGTA and 10 HEPES (titrated to pH 7.2 using KOH). For application of fluid flow, endothelium or a membrane patch was maneuvered to the exit of a capillary tube with tip diameter of 350 μm, out of which ionic (bath) solution flowed at 20 μL.s^{-1}. For pressure pulses, 0.2 s duration square pulses were applied to the patch pipette every 5 s using a High Speed Pressure Clamp HSPC-1 System (ALA Scientific Instruments, USA). Prior to the patch-recordings, freshly isolated endothelial cells were pre-treated with the inhibitors, the combination of inhibitors with lipids or vehicle control (0.25% DMSO) for 10 mins at room temperature. During the recordings, the inhibitors or the combination of inhibitor with lipid was still continuously present. The only exception was made for the experiments indicated in Figure 7 in which the lipids were acutely applied after the cells were pre-treated with the inhibitor alone for 10 minutes at room temperature.

Culture and Transfection of Human Umbilical Vein Endothelial Cells (HUVECs)

HUVECs were purchased from Lonza and cultured in Endothelial Cell Basal Medium (EBM-2) supplemented with 2% fetal calf serum and: 10 ng.mL^{-1} vascular endothelial growth factor (VEGF), 5 ng.mL^{-1} human basic fibroblast growth factor, 1 μg.mL^{-1} hydrocortisone, 50 ng.mL^{-1} gentamicin, 50 ng.mL^{-1} amphotericin B and 10 μg.ml^{-1} heparin (BulletKitTM, Lonza). Experiments were performed on cells from passage 2-5. HUVECs were transfected with 20 nM siRNA using Lipofectamine 2000 in OptiMEM (GIBCO) as per the manufacturer's instructions (Invitrogen). Medium was replaced after 4-5 hr and cells were used for experimentation 48 hr post-transfection. PIEZO1 siRNA was GCCUCGUGGUCUACAAGAUtt (Ambion), which was previously validated (Li et al., 2014). Non-targeting control siRNA was from Dharmacon.

PIEZO1 Tetracycline Inducible HEK293 Cell Line

HEK T-REx cells which overexpress human PIEZO1 on induction with tetracycline (P1-HEK cells) were as described previously (Evans et al., 2018; Rode et al., 2017). Expression was induced by treating the cells for 24 hr with 10 ng.mL^{-1} tetracycline (Sigma). Cells were cultured in Dulbecco's modified Eagle's medium-F12 GlutaMAX (Invitrogen, Paisley, UK) supplemented with 10% fetal calf serum (Sigma) and 1% penicillin/streptomycin (Sigma-Aldrich). Subsequently, cells were treated with 10 μg.mL^{-1} blasticidin and 200 μg.mL^{-1} zeocin (Invitrogen, Thermo Fisher Scientific) to select for stably transfected cells.

Intracellular Ca^{2+} Measurement

Measurements were made at room temperature on a FlexStation 3 (Molecular Devices, California) bench-top fluorometer controlled by Softmax Pro software v5.4.5. Cells were plated in clear 96-well plates (Corning, NY, USA) at a confluence of 90% 24 hours before experimentation. Cells were incubated with fura-2AM (2 μM) (Molecular ProbesTM) in SBS containing 0.01% pluronic acid (Thermo Fisher Scientific) for 60 min at 37°C. Cells were washed with SBS for 30 min at room temperature. Baseline fluorescence ratios were recorded before the addition of the compound solution to the cell plate after 60 s, with recording thereafter for a total of 300 s. The Standard Bath Solution (SBS) contained: NaCl 135 mM, KCl 5 mM, $MgCl_2$ 1.2 mM, $CaCl_2$ 1.5 mM, D-glucose 8 mM and HEPES 10 mM. pH was titrated to 7.4 using 4M NaOH.

PIEZO1 Channel Modeling

Structural data were obtained from the mouse cryo-EM structure PDB: 6B3R (Guo and MacKinnon, 2017). A total of 58 missing residues (~2.3% of the overall protein length) were added with MODELER (v 9.19) (Fiser and Sali, 2003; Sali and Blundell, 1993). These residues are 601-604, 876-879, 887-891, 1998-2014, 2066-2074, 2412-2423, 2457-2462 and 2547 (see Figure S7). The loop refinement tool was used to remove a knot in one chain between residues 2066-2074. The loop was selected out of 10 candidates according to the discrete optimized protein energy method (Shen and Sali, 2006). The final PIEZO1 mouse model does not comprises the first N-terminal 576 residues and residues 718-781, 1366-1492, 1579-1654, 1808-1951. Therefore, each chain is composed by five non-overlapping fragments: residues 577-717, 782-1365, 1493-1578, 1655-1807 and 1952-2547. The PIEZO1 model obtained was further energy minimized in vacuum with GROMACS 5.0.7 (Abraham et al., 2015) prior simulations.

Coarse-Grained Simulations

The PIEZO1 model obtained as described above was converted to a coarse-grained (CG) resolution and energy minimized. The CG molecular dynamics (CG-MD) simulations were performed using the Martini 2.2 force field (de Jong et al., 2013; Marrink et al., 2007) and GROMACS 5.0.7 (Abraham et al., 2015). In the Martini force field, there is an approximate 4:1 mapping of heavy atoms to coarse-grained particles. To model the protein secondary and tertiary structure an elastic network model with a cut-off distance of 7 Å was used. The elastic network restricts any major conformational change within the protein during the CG-MD simulations. This elastic network was used in a number of studies that showed good agreement with experimental data (Corradi et al., 2019; De Vecchis et al., 2019). Further, the model was inserted in a complex asymmetric bilayer using the INSert membrANE tool (Wassenaar et al., 2015). For the upper leaflet a concentration of 55% 1-palmitoyl-2-oleyl-phosphtidylcholine (POPC), 5% sphingomyelin, 20% 1-palmitoyl-2-oleyl-phosphtidylethanolamine (POPE), and 20% cholesterol, was used. For the lower leaflet a concentration of 50% POPC, 20% POPE, 5% sphingomyelin, 20% cholesterol, 5% 1-palmitoyl-2-oleyl-phosphtidylserine, and 5% phosphatidylinositol 4,5-bisphosphate, was used. Further simulations were performed substituting half of the sphingomyelin in the upper leaflet (i.e., 2.5%) with ceramide or by depleting all sphingomyelin (i.e., 5%) with ceramide. The systems were neutralized with 150 mM NaCl. The model was further energy minimized and subsequently equilibrated for 500 ns with the protein particles restrained (1000 $kJ.mol^{-1}.nm^{-2}$) to allow the membrane bilayer to equilibrate around the protein. After each equilibration, five unrestrained repeat simulations of 5 μs each were run for every system starting from different velocities. Both equilibration and production runs were performed at 323 K to be above the transition temperature of all the lipid species used and thus avoid non-fluid phases. Protein, lipids and solvent were separately coupled to an external bath using the V-rescale thermostat (Bussi et al., 2007) (coupling constant of 1.0). Pressure was semi-isotropically maintained at 1 bar (coupling constant of 1.0) with compressibility of 3×10^{-6} using the Berendsen (Berendsen et al., 1984) and the Parrinello-Rahman (Parrinello, 1981) barostats, for the equilibration and productions, respectively. Lennard-Jones and Coulombic interactions were shifted to zero between 9 and 12 Å, and between 0 and 12 Å, respectively.

Trajectory Analyses and Molecular Graphics

Root mean-square deviation (RMSD) and root mean-square fluctuation (RMSF) were performed using the g_rms and g_rmsf tools from the GROMACS package. The analysis suggested a stable structure (Figure S7). Molecular graphics were generated with the VMD 1.9.3 (Humphrey et al., 1996; http://www.ks.uiuc.edu/Research/vmd/) and data were plotted using Grace (https://plasma-gate.weizmann.ac.il/Grace/). For the calculation of dome depth, the simulation trajectory was fitted to the protein coordinates (reference structure). The coordinates of the CG phosphate particles in each frame of the fitted trajectory were extracted by a Python script. The phosphate particles were then used to separate the bilayer leaflets using a branching network algorithm. Briefly, this method started with a single phosphate particle, and identified the other phosphate particles which were within a cut-off distance (2 nm) of the starting particle. The cut-off distance was selected to be smaller than the separation between the bilayer leaflets. Phosphate particles identified in this way were grouped into the same leaflet as the starting residue. This process iterated repeatedly until no more new particles could be added, and the remaining particles were assumed to be part of the other leaflet. The process was then repeated starting in the other leaflet to validate the initial identification. For each leaflet, the depth of the dome was calculated as the difference between the surface level and the bottom of the dome. The surface level was taken to be the average z-coordinate for the phosphate particles with z-coordinate in excess of the 90th centile. The average was used here to minimize the effect of random fluctuation of the membrane. For the bottom of the dome, the z-coordinate of the phosphate particle with the absolute lowest z-coordinate was used. This was because the bottom of the dome was prone to far less fluctuation, being fixed to PIEZO1, which in turn was the fitting reference for the rest of the simulation. To calculate the 2-dimentional height map of the z-coordinate of the

CG phosphate particles in our simulations, CG phosphate particles from each leaflet were binned along the x and y axes for each frame; 75 bins for each axis. For each frame, the average z-coordinate of beads contained in each bin was calculated and stored in a matrix. The matrices of all frames were averaged to create the final height map, which was plotted using the PyPlot library. The code used was documented at: https://github.com/jiehanchong/membrane-depth-analysis.

QUANTIFICATION AND STATISTICAL ANALYSIS

Genotypes of mice were always blinded to the experimenter and mice were studied in random order determined by the genotype of litters. Data were generated in pairs (control mice and *Smpd3*$^{fro/fro}$ mice) and datasets compared statistically by independent t test without assuming equal variance. Paired t tests were used when comparing data before and after application of flow or a substance to the same membrane patch or cell. One-way ANOVA followed by Tukey posthoc test was used for comparing multiple groups. Statistical significance was considered to exist at probability (P) < 0.05 (* < 0.05, ** < 0.01, *** < 0.001). Where data comparisons lack an asterisk, they were not significantly different. The number of independent recordings is indicated by n and the number of mice per dataset by N (in total, murine data are from n = 278 recordings and N = 105 mice). For multi-well assays, the number of replicates is indicated by *N*. For amplitude histogram analysis the bin width was 0.05 pA. Descriptive statistics are shown as mean ± s.e.mean. Origin Pro software was used for data analysis and presentation.

Cell Reports, Volume *33*

Supplemental Information

Sphingomyelinase Disables Inactivation in Endogenous PIEZO1 Channels

Jian Shi, Adam J. Hyman, Dario De Vecchis, Jiehan Chong, Laeticia Lichtenstein, T. Simon Futers, Myriam Rouahi, Anne Negre Salvayre, Nathalie Auge, Antreas C. Kalli, and David J. Beech

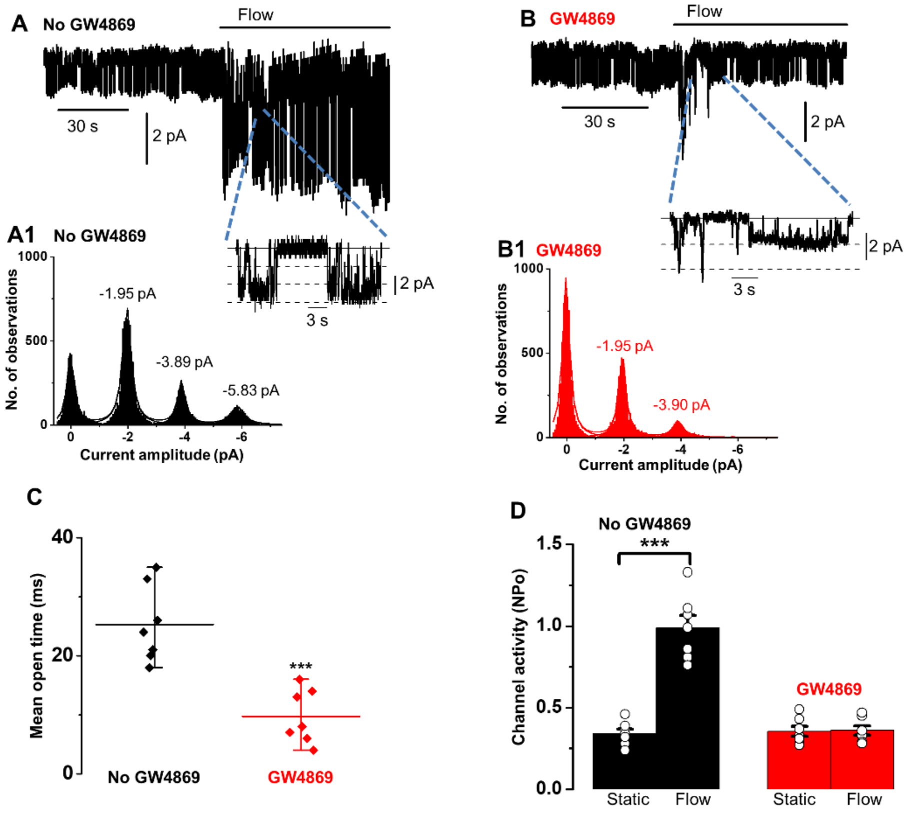

Figure S1

Figure S1. GW4869 Prevents Sustained Channel Activation by Flow. (**A**, **B**) Example traces for PIEZO1 channel currents in outside-out patches voltage-clamped at -80 mV. Constitutive channel activity was observed prior to application of flow at 20 µL.s^{-1}, which stimulated multiple channel openings. (**A**) Control condition: vehicle (0.25% DMSO). (**B**) Test condition: 10 µM GW4869. (**A1**, **B1**) Amplitude histograms for flow-evoked channel activity of (**A**) and (**B**). Current amplitudes for peaks of the fitted distributions are indicated. (**C**) Mean ± s.e.mean channel activity data for experiments of the type shown in (**A**, **B**): Control (n=7 recordings, N=3 mice); GW4869 (n=7, N=3). NP_o is the number of channels multiplied by the open probability of the channels. * *P* < 0.05 by Student's t-test. Related to Figure 3.

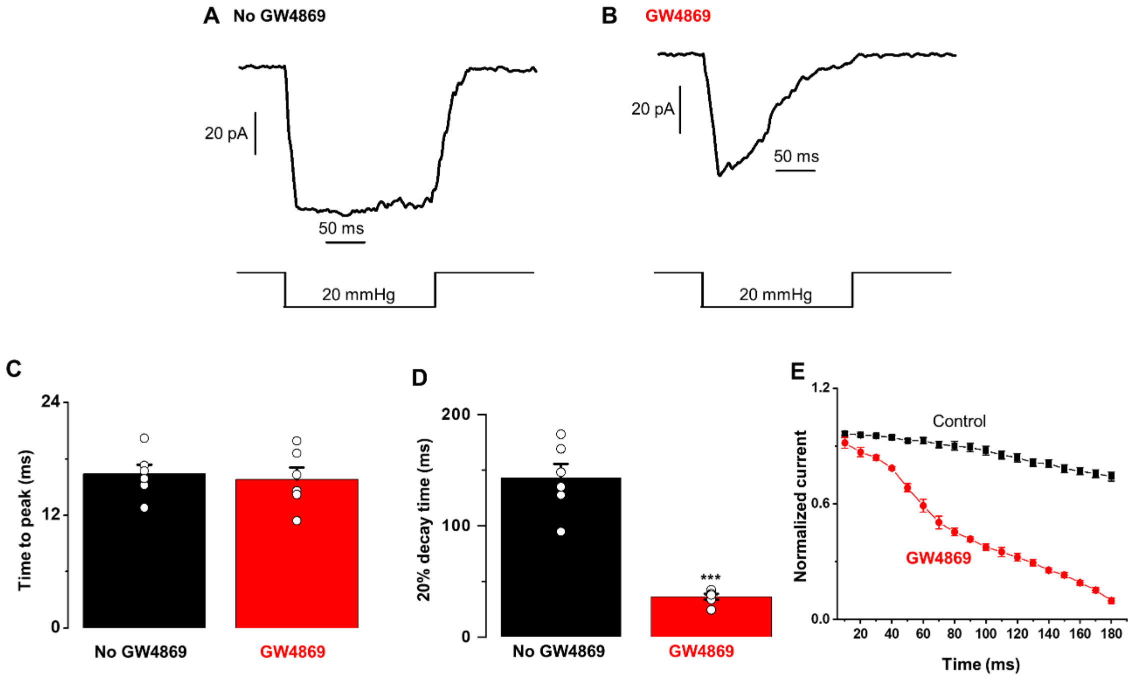

Figure S2

Figure S2. GW4869 Prevents Sustained Channel Activation By Pressure Step. (**A**, **B**) Example traces for macroscopic PIEZO1 channel currents in cell-attached patches voltage-clamped at -80 mV. Negative pressure steps of 20 mmHg were applied to the patch pipette. (**A**) Control condition: vehicle (0.25% DMSO). (**B**) Test condition: 10 µM GW4869. (**C**) Mean ± s.e.mean data for experiments of the type shown in (**A**, **B**), showing the time to reach peak inward current after first applying the pressure step: Control (n=6 recordings, N=3 mice); GW4869 (n=6 recordings, N=3 mice). (**D**) Mean ± s.e.mean data for experiments of the type shown in (**A**, **B**), showing the time for 20% decay of the current after reaching the peak: Control (n=6, N=3); GW4869 (n=6, N=3). *** $P < 0.001$ by Student's t-test. (**E**) As for (**A, B**) showing analysis of the current decay rate for: Control (n=6, N=3) and GW4869 (n=6, N=3) recordings. Related to Figure 4.

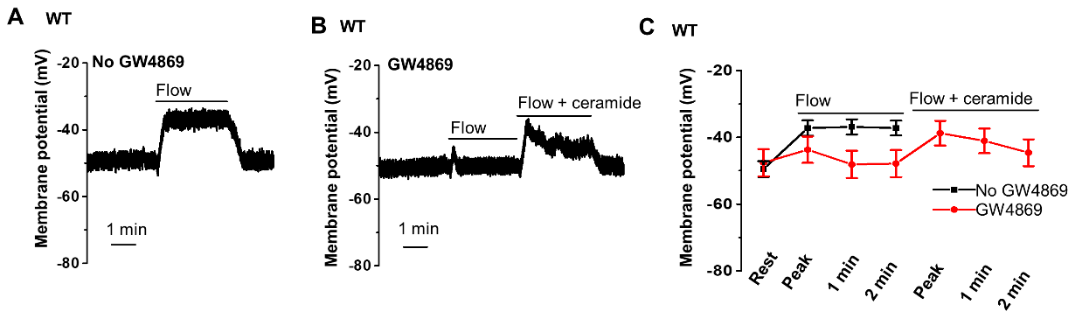

Figure S3

Figure S3. Wildtype Mouse Data For Comparison With Figure 5G-I. All data relate to membrane potential recorded in amphotericin whole-cell mode from freshly isolated endothelium of second-order mesenteric arteries. All data are from endothelium of wildtype (WT) mice. (**A**, **B**) Representative recordings showing responses to 20 μL.s^{-1} fluid flow in the absence and then presence of 10 μM ceramide and in the absence (**A**) and presence (**B**) of 10 μM GW4869. (**C**) Membrane potential mean ± s.e.mean data for experiments of the type shown in (**A**, **B**): n=6 recordings and N=3 mice for both groups. Related to Figure 5.

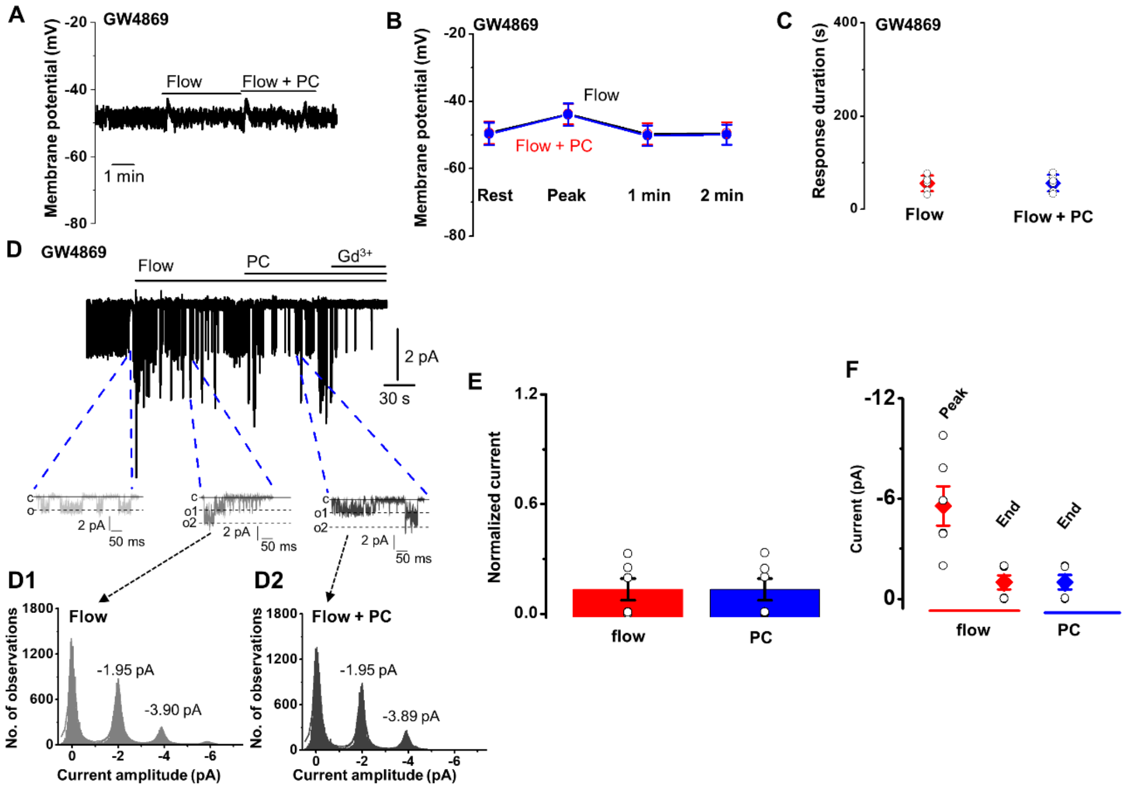

Figure S4

Figure S4. Phosphorylcholine (2-methacryloyloxyethyl PC) Has No Effect. (**A-C**) All data relate to membrane potential recorded in amphotericin whole-cell mode from freshly isolated endothelium of second-order mesenteric arteries. (**A**) Representative recording showing responses to 20 μL.s^{-1} fluid flow in the presence of 10 μM GW4869 in the absence and then presence of 10 μM PC. (**B**) Membrane potential mean ± s.e.mean data for experiments of the type shown in (**A**). (**C**) Response duration mean ± s.e.mean data for experiments of the type shown in (**A**). Original raw data points are superimposed. (**B**, **C**) n=6 recordings, N=3 mice. (**D-F**) All measurements were single channel current recordings from outside-out patches excised from freshly isolated endothelium of second-order mesenteric arteries. Holding potential was -80 mV. (**D**) Representative recording showing response to 20 μL.s^{-1} fluid flow in the presence of 10 μM GW4869 in the absence and then presence of 10 μM PC. Three sections of the traces are shown below on expanded time-scale. (**D1**, **D2**) Amplitude histograms for channel activity in flow and flow plus 2-methacryloyloxyethyl PC, as shown in (**D**). Current amplitudes for peaks of the fitted distributions are indicated. (**E**) For experiments of the type shown in (**D**), all data points and mean ± s.e.mean for the channel currents at the end of recordings with the application of PC (before the addition of Gd^{3+}) normalized to peak currents evoked by flow (n=6 recordings, N=3 mice). (**F**) The values of peak current or the current at the end of recording for each patch shown in (**D**). Related to Figure 6.

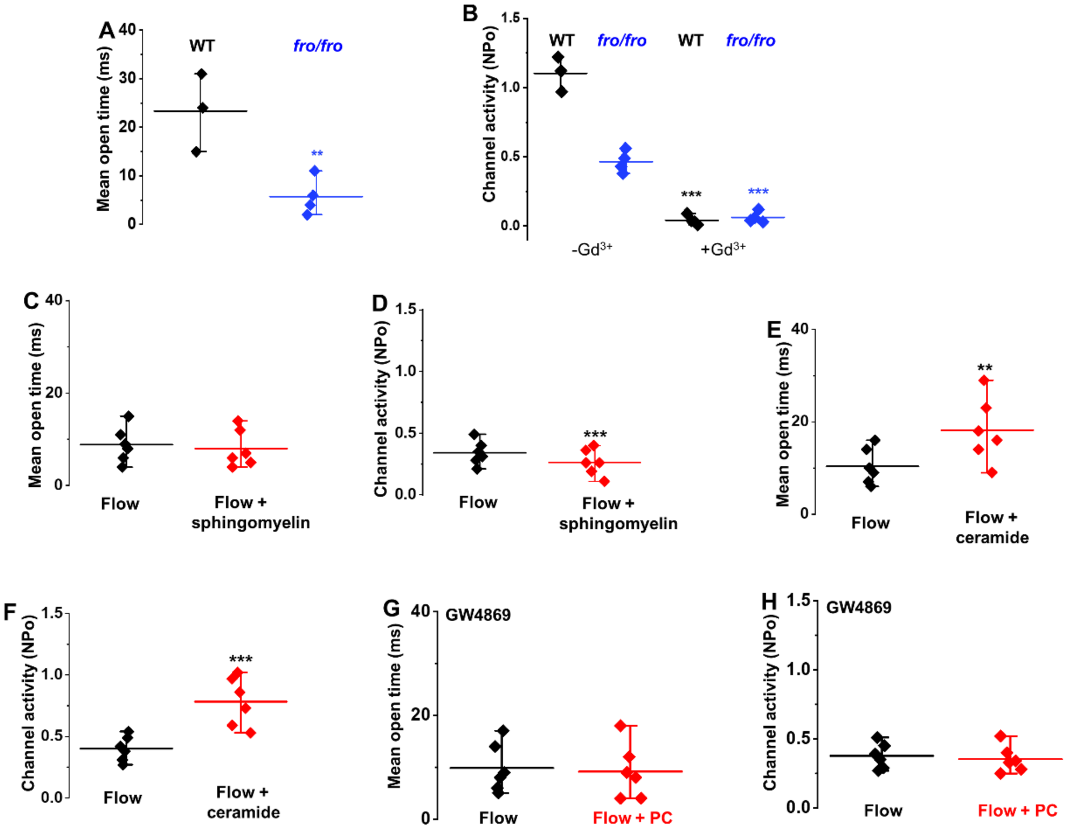

Figure S5

Figure S5. Mean Open Time And Channel Open Probability For The Experiments In Endothelial Cells From WT/ *fro/fro* Mice Or Pre-Treated With Different Lipids. (**A**) Mean open time for experiments of the type shown in **Figure 3A and Figure 3B**, all data points and mean ± s.e.mean for the channel open time 20 s after the initiation of flow (i.e. the sustained response) (WT n=3 recordings, N=3 mice, *fro/fro* n=4, N=3). ** $P < 0.01$. (**B**) Channel open probability (NPo) for experiments of the type shown in **Figure 3** (**A**, **B**, **E**, **F**), all data points and mean ± s.e.mean for channel activity represented by NP_o (channel number multiplied by open probability) measured 20 s after the initiation of flow. Without Gd^{3+} (- Gd^{3+}): WT n=3, N=3; *fro/fro* n=4, N=3. With Gd^{3+} (+ Gd^{3+}): WT n=4, N=3; *fro/fro* n=4, N=3. *** $P < 0.001$. (**C**) Channel open time mean ± s.e.mean data for experiments of the type shown in **Figure 6** (**A**): n=6 recordings, N=3 mice. (**D**) Mean ± s.e.mean channel activity described as NP_o (channel number multiplied by open probability) for experiments of the type shown in **Figure 6** (**A**): n=6 recordings, N=3 mice. (**E**) Channel open time mean ± s.e.mean data for experiments of the type shown in **Figure 6** (**D**): n=6 recordings, N=3 mice. (**F**) Mean ± s.e.mean channel activity described as NP_o for experiments of the type shown in **Figure 6** (**D**): n=6 recordings, N=3 mice. (**G**) Channel open time mean ± s.e.mean data for experiments of the type shown in **SI Figure S4** (**D**): n=6 recordings, N=3 mice. (**H**) Mean ± s.e.mean channel activity described as NP_o (channel number multiplied by open probability) for experiments of the type shown in **SI Figure S4** (**D**): n=6 recordings, N=3 mice. Original raw data points are superimposed. Related to Figure 3.

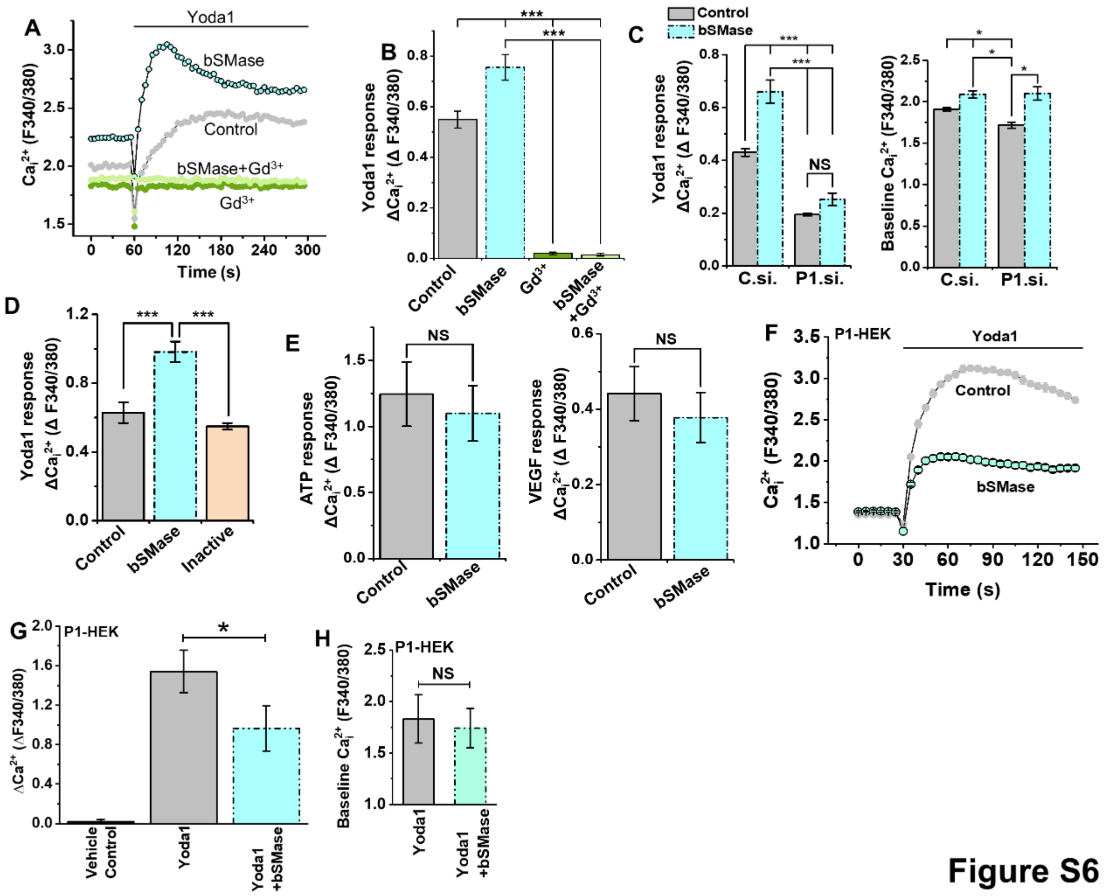

Figure S6

Figure S6. Bacterial Sphingomyelinase Enhances Endothelial PIEZO1 Function But Inhibits PIEZO1 Over-Expressed In HEK 293 Cells. When we first began this project we tested the effects of exogenous bacterial sphingomyelinase (bSMase) on PIEZO1-mediated Ca^{2+} signals evoked by the chemical activator, Yoda1, in cultured human umbilical vein endothelial cells (HUVECs) or HEK 293 cells over-expressing human PIEZO1. Previous studies had described how addition of exogenous sphingomyelinase can modify gating properties of voltage-gated ion channels (Combs et al., 2013). HUVECs were previously shown to express native PIEZO1 (Evans et al., 2018; Li et al., 2014). Data are intracellular Ca^{2+} measurements from HUVECs or from HEK T-REx™ cells overexpressing human PIEZO1 on induction with tetracycline (P1-HEK cells). (**A**) Example time-series traces showing effects of 1 μM Yoda1 on intracellular Ca^{2+} indicated by F340/380 in cells in a single 96-well plate without pre-treatment (Control) or after pre-treatment for 30 min at 37 ˚C with 0.5 $U.mL^{-1}$ bSMase or 100 μM gadolinium chloride (Gd^{3+}) or both. (**B**) As for (**A**) but plotted as the peak change in intracellular Ca^{2+} (ΔCa^{2+}_i) above baseline in response to Yoda1 and showing mean ± s.e.mean data for multiple independent experiments (n/*N*=3/4). (**C**) Mean ± s.e.mean data for ΔCa^{2+}_i in response to 1 μM Yoda1 (left) and baseline Ca^{2+} indicated by basal F340/380 in the same experiments (n/*N*=3/4). C.si. was control siRNA and P1.si. PIEZO1 siRNA. (**D**) Similar to (**B**) but comparing the effect of 30 min pre-treatment with 0.5 $U.mL^{-1}$ bSMase or the same concentration of heat-inactivated bSMase on the effect of 1 μM Yoda1 (n/*N*=3/4). (**E**) Similar to (**B**) but in which Ca^{2+} responses were evoked by 20 μM ATP or 30 $ng.mL^{-1}$ vascular endothelial growth factor (VEGF) in place of Yoda1 (n/*N*=3/4 each). (**F**) Example time-series traces showing effects of 1 μM Yoda1 on intracellular Ca^{2+} indicated by F340/380 in cells without pre-treatment (Control) or after pre-treatment for 30 min at 37 ˚C with 0.5 $U.mL^{-1}$ bSMase. In the absence of exogenous PIEZO1 these cells show no Ca^{2+} response to Yoda1(Evans et al., 2018). (**G**) As for (**F**) but plotted as the peak change in intracellular Ca^{2+} (ΔCa^{2+}_i) above baseline in response to the vehicle control for Yoda1 (DMSO), 1 μM Yoda1 or 1 μM Yoda1 after pre-treatment with bSMase, showing mean ± s.e.mean data for multiple independent experiments (n/*N*=3/4). (**H**) Mean ± s.e.mean data for baseline Ca^{2+} indicated by basal F340/380 in the same experiments as (**G**). *** $P < 0.01$, * $P < 0.05$, NS:not significantly different. Related to Figures 3 and 4.

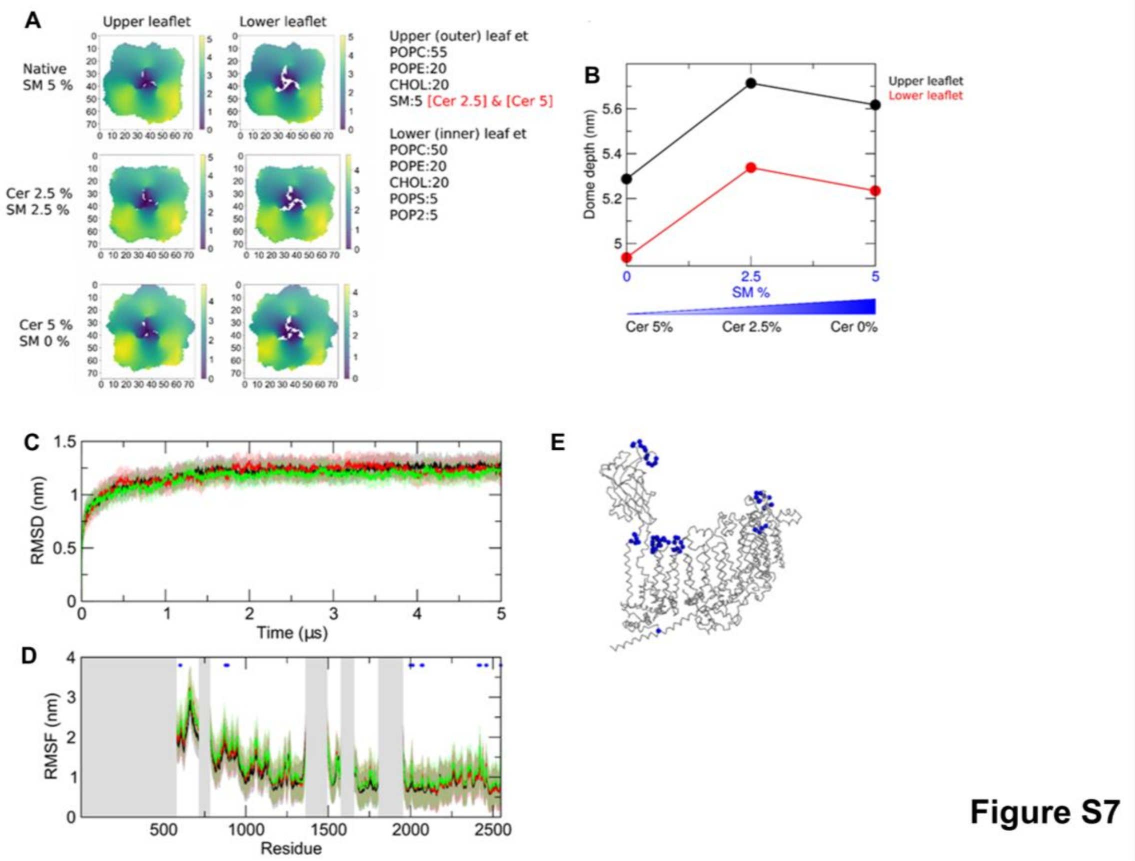

Figure S7. Potential Molecular Mechanism Underlying Regulation Of Piezo1 By Ceramide And Sphingomyelin In Endothelial Cells. To investigate whether the dome could be affected by changes in sphingomyelin and ceramide concentration, we performed molecular dynamics simulations in which the 3D structure of PIEZO1 (Guo and MacKinnon, 2017) was inserted in model asymmetric membranes that resemble the endothelial plasma membrane (Takamura et al., 1990). We included different concentrations of sphingomyelin and ceramide: 5% sphingomyelin, 5% ceramide and a combination at 2.5% each. In all simulations, PIEZO1 created a dome in the membrane with a trilobed topology in good agreement with previous studies (Chong et al., 2019). However, the depth of the dome was lower in the systems in which 5% ceramide was added. Impact of sphingomyelin and ceramide on the PIEZO1 channel dome depth is shown in (A) and (B). Stability and fluctuation analyses of the PIEZO1 structure during the molecular dynamics simulations are shown in (C), (D) and (E). (**A**) Height map of the z-coordinate of the CG phosphate particles corresponding to the upper (left) and lower (right) leaflet, averaged across all repeat simulations of the same simulation system. This analysis is shown for the 3 different simulation systems with different concentrations of sphingomyelin and ceramide. (**B**) Average dome depth of the upper and lower leaflets in the 3 simulation systems. With 2.5% sphingomyelin and 2.5% ceramide, the dome reached maximum depth of 5.71 ± 0.00778 nm and 5.34 ± 0.00847 nm for the upper and lower leaflets. With 5% sphingomyelin the depth was 5.62 ± 0.00899 nm and 5.23 ± 0.00963 nm for the upper and lower leaflets, whereas with 5% ceramide it was 5.29 ± 0.00708 nm and 4.94 ± 0.00767 nm. All phospholipids and the sphingomyelin remained in the leaflet of their origin at the beginning of the simulations. Ceramide and cholesterol were seen to flip-flop between the leaflets. (**C**) Root mean square deviation (RMSD) of the protein as a function of time in the simulated systems. The RMSD of the protein for the simulations with the native bilayer is shown in black, for the bilayer with 2.5% ceramide is shown in red, and for the membrane with 5% ceramide is shown in green. (**D**) Root mean-square fluctuation (RMSF) of the protein calculated in the simulated systems. The color code is the same as in (**C**). Blue spheres show the residues that are not present in the PIEZO1 cryo-EM structure that have been added during the modelling (see STAR Methods). (**E**) Last frame of one of the coarse-grained simulations showing a single chain from the PIEZO1 structure (gray). The residues in blue are the residues that are missing from the cryo-EM structure that were added during the modelling stage (see STAR Methods). Related to Figure 7.
